# Deep Learning Analysis of COVID-19 Vaccine Hesitancy and Confidence Expressed on Twitter in 6 High-Income Countries: Longitudinal Observational Study

**DOI:** 10.2196/49753

**Published:** 2023-11-06

**Authors:** Xinyu Zhou, Suhang Song, Ying Zhang, Zhiyuan Hou

**Affiliations:** 1 School of Public Health Fudan University Shanghai China; 2 Global Health Institute Fudan University Shanghai China; 3 Department of Biostatistics Yale School of Public Health New Haven, CT United States; 4 Department of Health Policy and Management College of Public Health University of Georgia Athens, GA United States

**Keywords:** COVID-19 vaccine, hesitancy, confidence, social media, machine learning

## Abstract

**Background:**

An ongoing monitoring of national and subnational trajectory of COVID-19 vaccine hesitancy could offer support in designing tailored policies on improving vaccine uptake.

**Objective:**

We aim to track the temporal and spatial distribution of COVID-19 vaccine hesitancy and confidence expressed on Twitter during the entire pandemic period in major English-speaking countries.

**Methods:**

We collected 5,257,385 English-language tweets regarding COVID-19 vaccination between January 1, 2020, and June 30, 2022, in 6 countries—the United States, the United Kingdom, Australia, New Zealand, Canada, and Ireland. Transformer-based deep learning models were developed to classify each tweet as intent to accept or reject COVID-19 vaccination and the belief that COVID-19 vaccine is effective or unsafe. Sociodemographic factors associated with COVID-19 vaccine hesitancy and confidence in the United States were analyzed using bivariate and multivariable linear regressions.

**Results:**

The 6 countries experienced similar evolving trends of COVID-19 vaccine hesitancy and confidence. On average, the prevalence of intent to accept COVID-19 vaccination decreased from 71.38% of 44,944 tweets in March 2020 to 34.85% of 48,167 tweets in June 2022 with fluctuations. The prevalence of believing COVID-19 vaccines to be unsafe continuously rose by 7.49 times from March 2020 (2.84% of 44,944 tweets) to June 2022 (21.27% of 48,167 tweets). COVID-19 vaccine hesitancy and confidence varied by country, vaccine manufacturer, and states within a country. The democrat party and higher vaccine confidence were significantly associated with lower vaccine hesitancy across US states.

**Conclusions:**

COVID-19 vaccine hesitancy and confidence evolved and were influenced by the development of vaccines and viruses during the pandemic. Large-scale self-generated discourses on social media and deep learning models provide a cost-efficient approach to monitoring routine vaccine hesitancy.

## Introduction

The COVID-19 pandemic has been around for a long time, with impacts on all sectors of society [1]. Sustaining a high level of immunity through vaccination and booster shots among the public, especially the at-risk population, is a key strategy for controlling the impact of the pandemic. COVID-19 vaccine hesitancy has gradually become a critical barrier to the uptake of the vaccine [2]. Although a high level of vaccination rate was observed in many sectors of the world, the unequal global distribution of vaccination and the formation of antivaccine groups on platforms such as Twitter continue to contribute to localized transmissions of COVID-19 and public health burden [3-5]. Therefore, understanding and maintaining the public’s adherence to vaccination still pose a challenge for policy makers worldwide [6-11]. Current evidence reported a number of factors associated with COVID-19 vaccine hesitancy, including sociodemographic factors [4,6] and vaccine confidence, providing strategies to combat vaccine hesitancy in local contexts [7,12,13]. On the other hand, ongoing monitoring of the national and subnational trajectory of COVID-19 vaccine hesitancy in multiple countries could still offer support in designing tailored policies on improving vaccine uptake.

As a conventional approach, previous studies conducted surveys to investigate COVID-19 vaccine hesitancy, confidence, and the associated barriers [8,9]. However, the high cost of surveys restricts the ability to draw conclusions about the longitudinal changes in vaccine hesitancy and confidence over time. In recent years, social media has become a popular platform for individuals to express their experiences and viewpoints on various topics, including vaccination. Thus, social media mining has gained recognition as a supplement for understanding and responding to public attitudes and behaviors, particularly during public health emergencies like the COVID-19 pandemic [14-16]. The vast number of posts on social media platforms can provide extensive and up-to-date longitudinal data, starting from the onset of the pandemic to the present. Those social media data can assist in monitoring the trajectory of public hesitancy and confidence toward vaccines throughout the progress of COVID-19 vaccine development, authorization, and deployment, which may support policy making, health communication strategies, and the prediction of the responses to new vaccines [14]. Besides, recent advances in deep learning models reached state-of-the-art performance in various natural language processing (NLP) tasks, making it possible to predict vaccine hesitancy and confidence using massive social media data close to real time [17-19]. Some previous studies monitored the sentiments and discussion topics around COVID-19 vaccines using social media data [20-23]. However, most of them were about a specific country, covering a relatively short time period, or did not fine-tune state-of-the-art deep learning models using task-specific manually annotated data sets.

Thus, our study aimed to (1) monitor the national and subnational spatiotemporal trends in COVID-19 vaccine hesitancy and confidence in 6 high-income countries during the entire pandemic period using deep learning analysis of Twitter data; (2) examine the disparities in vaccine hesitancy and confidence by country, manufacturer, and states within a country; and (3) identify the potential sociodemographic factors associated with the hesitancy and confidence in COVID-19 vaccination.

## Methods

### Ethical Considerations

This study is exempt from ethics approval because it used deidentified Twitter data, which is available to the public, does not include identifiable health information, and ensures anonymity.

### Data Collection

We collected COVID-19 vaccine–related tweets and identified the tweets in 6 English-language high-income countries, namely the United States, the United Kingdom, Canada, Australia, New Zealand, and Ireland. Similar to previous studies [24,25], data were collected using TweetScraper, a Python tool for collecting Twitter search data [26]. Geo-locations were identified based on the user’s profile location and Carmen [27], a tool for geolocating tweets. Duplicated tweets and tweets without standardized locations or outside the 6 countries were excluded. In total, we collected 5,257,385 English-language tweets containing keywords “(covid OR coronavirus OR covid19 OR covid-19) AND (vaccine OR vaccination)” which were posted on Twitter in the 6 countries from January 1, 2020, through June 30, 2022.

### Fine-Tuning Deep Learning Model for Annotating COVID-19 Vaccine–Related Tweets

Proposed in 2018, BERT (Bidirectional Encoder Representations from Transformers) is a groundbreaking deep learning model for NLP. It can perform a wide array of NLP tasks, such as question answering, language generation, and text classification [18]. BERT was pretrained with the Toronto Book Corpus and Wikipedia data set, which contains billions of pieces of text and provides the model with a comprehensive understanding of language [18].

While the BERT model is pretrained on the text data for more than a billion words, the corpus mainly consists of information on mixed domains, with no inclination to any subdomains [18]. In order to have better language interpretation results on COVID-19–related subdomains on Twitter, we adopt the COVID-Twitter-BERT (CT-BERT) model, which is specially pretrained on top of BERT for COVID-19–related tweets [19]. The CT-BERT model adopts the same model structure as BERT_LARGE_, while it was further pretrained on a corpus of 160 million tweets about COVID-19 and evaluated with 4 Twitter data sets—COVID-19 category, vaccine sentiment, maternal vaccine stance, and sentiment evaluation (SemEval-2016 task 4 subtask A), along with a stand-alone data set specialized for sentiment analysis [19]. It improves BERT_LARGE_’s marginal performance in reviewing COVID-19–related tweets by 9%-26% [19].

To use CT-BERT for evaluating COVID-19 vaccine hesitancy and confidence on Twitter, it needs to be further finetuned using a manually annotated data set on this topic. Our research team manually labeled 8073 tweets on COVID-19 vaccines. Each tweet was annotated by 2 annotators according to the framework of vaccine hesitancy proposed by the World Health Organization [28], and a third annotator resolved their disagreements. We finetuned CT-BERT models using 8073 tweets to generalize our analysis to all COVID-19 vaccine–related tweets we collected [16,29]. These initial, manually labeled tweets were divided into a training set (80%), a development set (10%), and a test set (10%). With the training set and development set, hyperparameters are chosen, and deep learning models are finetuned. The performance of the finetuned deep learning models was then evaluated with the test set.

### Deep Learning Prediction of COVID-19 Vaccine Hesitancy and Confidence

With the deep learning models, we first identified the tweets sent most likely by humans, where the models attained a performance with a precision of 0.89 and an *F*_1_-score of 0.86. A total of 3,348,746 tweets sent most likely by humans were identified and contained country-level geo-locations. Among these tweets, 2,601,672 tweets contain state-level geo-locations. The deep learning models further labeled them with the 4 predefined categories as the outcome variables of interest in our study concerning hesitancy and confidence in COVID-19 vaccination. Although our manually annotated data set was labeled into more categories, to ensure optimal model performance, we analyzed the following four categories in this study using deep learning: (1) intent to accept COVID-19 vaccination (precision=0.88, *F*_1_-score=0.86), (2) intent to reject COVID-19 vaccination *(*precision=0.78, *F*_1_-score=0.75), (3) belief that COVID-19 vaccines are effective (precision=0.81, *F*_1_-score=0.73), and (4) belief that COVID-19 vaccines are unsafe (precision=0.86, *F*_1_-score=0.75). Each tweet could contain 1 label, multiple labels, or no label at all. (1) Vaccine acceptance and (2) vaccine rejection are mutually exclusive with each other, whereas (3) vaccine effectiveness and (4) vaccine unsafe are not mutually exclusive with any other categories. Table S1 in Multimedia Appendix 1 presents the annotation categories and prediction performance of deep learning models for each category, and Table S2 in Multimedia Appendix 1 displays the number of tweets in each category and hyperparameters for model fine-tuning.

### Statistical Analysis

We first measured the individual-level vaccine hesitancy and confidence using the average deep learning prediction of all his or her tweets. For example, if an individual sent 2 tweets on COVID-19 vaccines during January 2022, among which 1 tweet indicates acceptance of vaccine, then his or her acceptance in January 2022 would be 50%. If he or she sent another 2 tweets indicating vaccine acceptance in February 2022, his or her vaccine acceptance would be 100% during February 2022, and his or her overall vaccine acceptance would be 75% during January-February 2022.

The spatiotemporal trends were then calculated as the average of all individuals (in a specific time period, place, or mentioning a specific vaccine manufacturer). For example, 100 people in the United Kingdom sent tweets on COVID-19 vaccines during January 2022; 60% of them expressed 100% acceptance toward COVID-19 vaccines, and 40% of them expressed 0% acceptance toward COVID-19 vaccines, then vaccine acceptance in the United Kingdom in January 2022 would be 60%×100% + 40%×0% = 60%. Due to data insufficiency during the early pandemic, January and February 2020 were not included in the temporal trend analysis. Vaccine manufacturers mentioned in tweets were detected with a keyword matching strategy using the keywords in Table S3 in Multimedia Appendix 1. The “overall” temporal trends are the country-level average of the 6 high-income countries.

To explore the variation within a country and the factors associated with hesitancy and confidence in COVID-19 vaccination, we conducted a state-level analysis in the United States, as there are sufficient data (1,812,398 tweets sent by 700,773 users) available for the analysis there. Bivariate and multivariable linear regressions were further used to examine the sociodemographic factors associated with hesitancy and confidence in COVID-19 vaccination across the states in the United States. The sociodemographic variables included political affiliation (Republican as the reference or Democrat party) [30], population density (number of people per square mile), percentage of people aged ≥65 years, and log-transformed gross domestic product per capita [31]. Current evidence shows that Democrats were estimated to have a higher vaccination rate, lower hesitancy toward COVID-19 vaccination, and fewer COVID-19 cases and deaths [6,32-35]. Population density was estimated to be associated with risks of infection, with a higher density catalyzing the spread of COVID-19 [36], which may lead to a higher willingness to COVID-19 vaccination. Older adults are more likely to be at higher risks for severe COVID-19 cases [37], and policies were encouraging the aged people to take COVID-19 vaccination, thus, more older adults may be associated with a higher acceptance rate and a lower rejection rate. Higher socioeconomic status (ie, greater gross domestic product per capita) has been reported to be associated with higher vaccination coverage [38]. Table S4 in Multimedia Appendix 1 describes state-level sociodemographic characteristics. For multivariable linear regressions, Model 1 included all sociodemographic variables, and Model 2 additionally adjusted belief in effectiveness and unsafety of COVID-19 vaccines. A variance inflation factor was estimated in each model, and every variance inflation factor was less than 10, indicating that multicollinearity didn’t exist. All analyses were performed with Python (version 3; Python Software Foundation), except for the factor analysis being carried out with STATA/SE (version 17; Stata Corporation).

## Results

**Figure 1:**
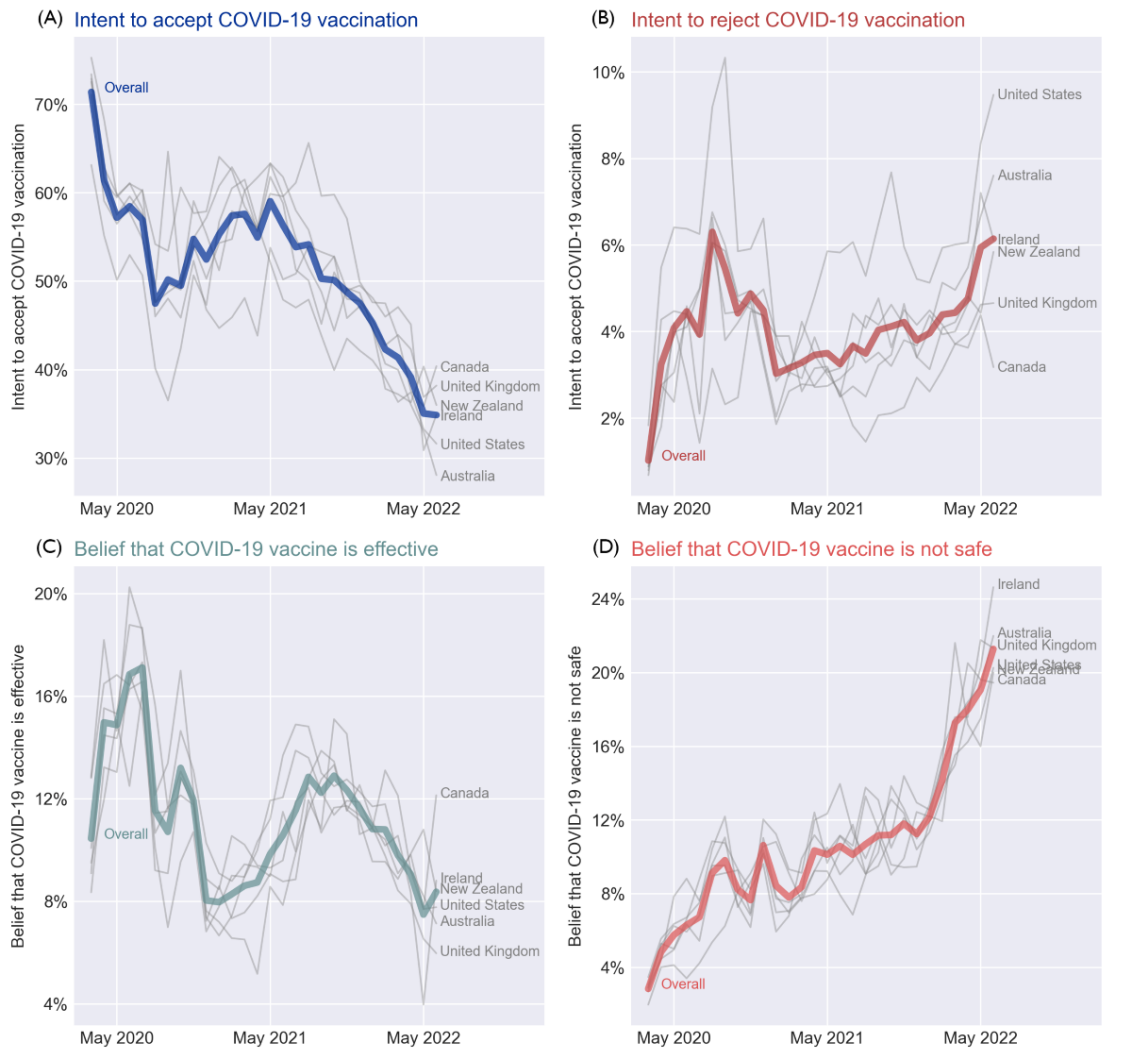
Temporal trends in COVID-19 vaccination hesitancy and confidence, March 2020-June 2022.

Figure 1 shows the temporal trends in COVID-19 vaccine hesitancy and confidence according to the prediction of deep learning models. Similar trends in the 6 countries were observed from March 2020 to June 2022. The average vaccine acceptance among the 6 countries decreased from 71.38% of 44,944 tweets in March 2020 to 47.46% of 47,327 tweets in August 2020. Subsequently, while there were some fluctuations, the rate slowly rose to 59.03% of 153,419 tweets in May 2021. This uptick coincided with the period when the results from clinical trials of COVID-19 vaccines were being published, showcasing the high efficacy and safety of the vaccines (Figure 1A). Notably, a slight dip was observed in April 2021 (54.93% of 216,667 tweets), coinciding with an adverse event reported on April 13 about a rare and severe type of blood clot experienced by 6 US women after receiving the Johnson & Johnson vaccine [39]. After May 2021, with the new variants of SARS-CoV-2 (eg, Delta in May 2021 and Omicron in November 2021) occurring and spreading across the globe [40,41], vaccine acceptance rate continuously decreased to 34.85% of 48,167 tweets in June 2022. Meanwhile, the trends in rejection rate basically mirrored the inverse acceptance rate’s trajectory (Figure 1B). The rejection rate increased from 1.02% of 44,944 tweets in March 2020 to 6.30% of 47,327 tweets in August 2020, and then fluctuated downward to 3.50% of 153,419 tweets in May 2021, ascending again to 6.14% of 48,167 tweets in June 2022.

The trend regarding the belief that COVID-19 vaccines is effective basically paralleled the trajectory in vaccine acceptance rate, but with a time lag (Figure 1C). After reaching the peak in July 2020 (17.11% of 49,176 tweets), the belief in vaccine effectiveness started to wane, reaching its lowest level of 7.96% of 239,882 tweets in January 2021, which was approximately 5 months after the nadir in the vaccine acceptance rate observed in August 2020. The belief in vaccine effectiveness continuously rose from January 2021 to October 2021, when the country-level average reached 12.89% of 150,128 tweets. As the Omicron variant emerged and spread in November 2021, the belief in vaccine effectiveness started to decline, dropping to 7.48% of 37,842 tweets in May 2022. On the other hand, throughout this study’s period, the belief that COVID-19 vaccines are not safe continuously rose by 7.49 times, from 2.84% of 44,944 tweets in March 2020 to 21.27% of 48,167 tweets in June 2022.

**Figure 2:**
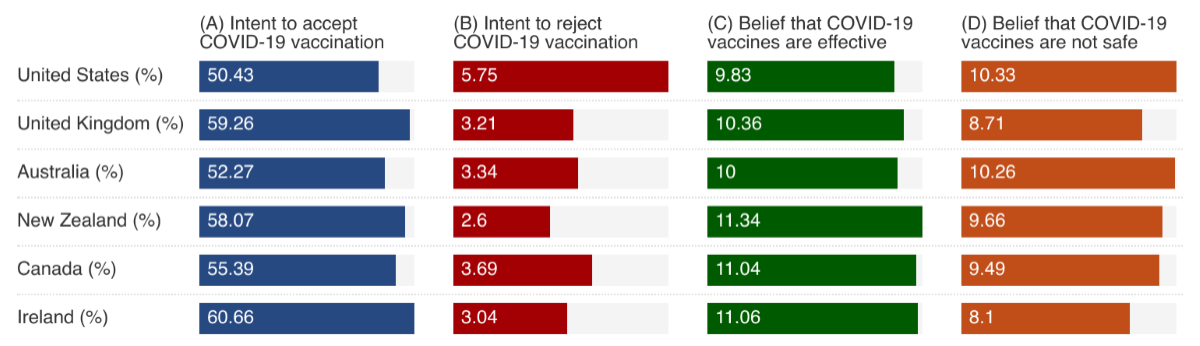
The prevalence of hesitancy and confidence in COVID-19 vaccination by country.

Figure 2 compares the prevalence of hesitancy and confidence in COVID-19 vaccination across the 6 countries. The overall vaccine acceptance was slightly different across the 6 countries, with the highest acceptance rate in Ireland (60.66% of 46,732 tweets) and the lowest rate at 50.43% of 2,228,907 tweets in the United States (Figure 2A). In contrast, the highest rejection rate was observed in the United States (5.75% of 2,228,907 tweets), while the other 5 countries exhibited similar rejection rates, hovering around 3% (Figure 2B). Meanwhile, the 6 countries had similar rates of belief in vaccine effectiveness (ranging from 9.83% of 2,228,907 tweets in the United States to 11.06% of 46,732 tweets in Ireland) and vaccine unsafety (ranging from 8.10% of 46,732 tweets in Ireland to 10.33% of 2,228,907 tweets in the United States; Figures 2C and D).

Variations in hesitancy and confidence toward COVID-19 vaccines by different manufacturers were observed across countries (Table 1). In Ireland, the United States, and Canada, Moderna and Pfizer vaccines reached higher acceptance rates (>50%), whereas Australia and New Zealand recorded lower acceptance rates (about 40%). The acceptance rate of the AstraZeneca vaccine was the highest in the United Kingdom (50.48% of 9185 tweets), surpassing the other 5 countries by 6%-19%. The Johnson & Johnson vaccine was most accepted in Ireland (60.04% of 92 tweets), marking it 15%-29% higher than the other 5 countries. Patterns in rejection rates and confidence toward vaccine safety and effectiveness were aligned with those in the acceptance rates.

**Table 1:** The prevalence of hesitancy and confidence in COVID-19 vaccination by country and vaccine manufacturer. Some statistics were calculated based on an insufficient amount of data.

Country		Pfizer	Moderna	AstraZeneca	Johnson & Johnson
**Intent to accept COVID-19 vaccination, %**					
	United States	49.58	53.13	33.40	37.10
	United Kingdom	49.23	45.52	50.48	44.74
	Australia	43.60	39.51	32.66	30.91^a^
	New Zealand	42.73	43.01^a^	31.11^a^	44.44^a^
	Canada	49.38	52.52	44.49	36.32
	Ireland	55.64	56.99	40	60.04^a^
**Intent to reject COVID-19 vaccination, %**					
	United States	3.06	2.29	4.63	4.46
	United Kingdom	2.26	2.31	1.56	3.07
	Australia	2.11	1.83	2.34	0^a^
	New Zealand	1.68	0.22^a^	2.67^a^	11.11^a^
	Canada	2.37	1.84	2.55	3.14
	Ireland	1.45	1.56	2.65	0.43^a^
**Belief that COVID-19 vaccines are effective, %**					
	United States	12.30	13.47	9.56	9.45
	United Kingdom	10.72	12.18	12.32	10.04
	Australia	8.24	7.99	9.36	1.82^a^
	New Zealand	9.61	15.23^a^	5.33^a^	0^a^
	Canada	11.49	11.45	12.88	8.55
	Ireland	11.84	12.28	8.30	16.03^a^
**Belief that COVID-19 vaccines are not safe, %**					
	United States	13.38	13.70	23.95	22.70
	United Kingdom	15.04	15.64	14.56	15.98
	Australia	15.41	16.29	18.43	18.18^a^
	New Zealand	17.12	21.04^a^	22.53^a^	33.33^a^
	Canada	14.88	13.42	16.59	24.08
	Ireland	14.30	13.09	18.28	8.12^a^

^a^Based on an insufficient amount of data (less than 100 Twitter users).

**Figure 3:**
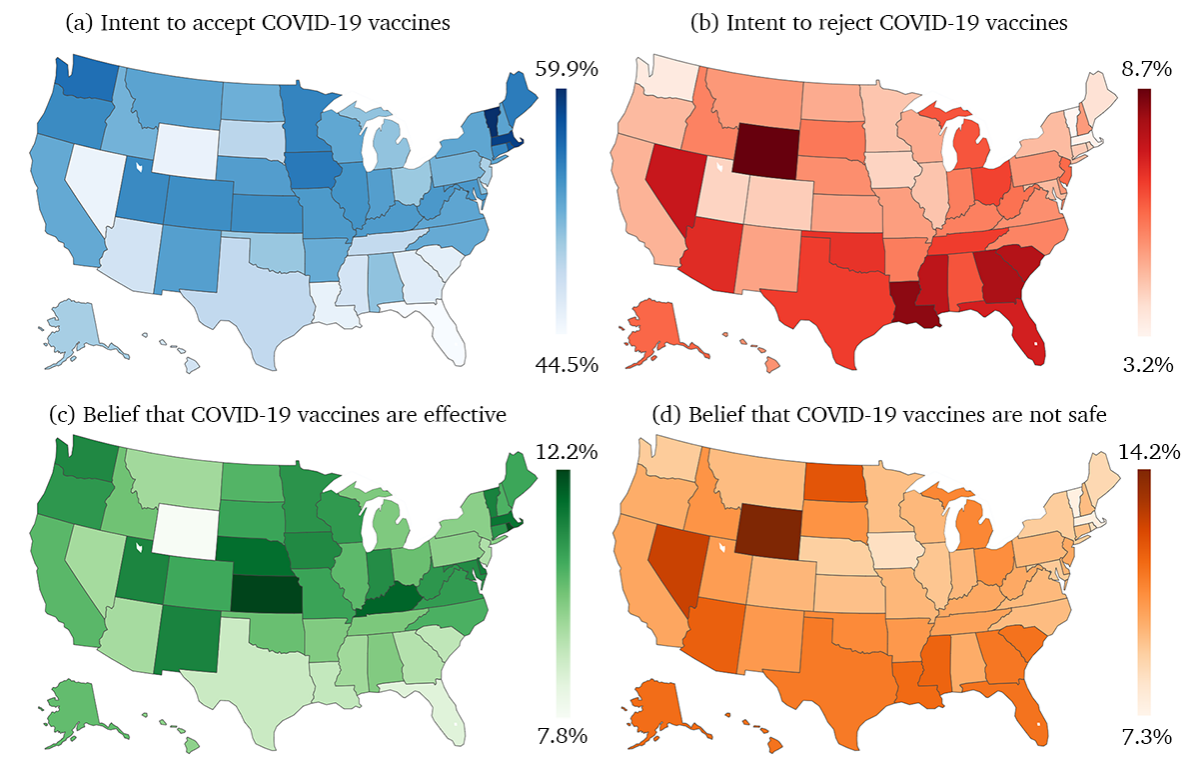
The prevalence of hesitancy and confidence in COVID-19 vaccination across the states in the United States.

In the United States, the prevalence of hesitancy and confidence in COVID-19 vaccination varied across the states (Figure 3). The acceptance rate of COVID-19 vaccination ranged from 44.50% to 59.90% across the states. Generally, northern states reported higher acceptance rates, whereas southern states showed lower acceptance. Specifically, Florida (44.50% of 121,996 tweets), Nevada (45.41% of 22,045 tweets), and Wyoming (45.53% of 1495 tweets) recorded the lowest acceptance rates. In contrast, Vermont (59.90% of 2903 tweets), Massachusetts (58.88% of 47,894 tweets), and the District of Columbia (57.81% of 53,627 tweets) had the highest acceptance rates (Figure 3A). States with higher acceptance rates typically have lower rejection rates (Figure 3B). Furthermore, confidence in COVID-19 vaccination mirrored the distribution of vaccine hesitancy (Figures 3C and D).

Table 2 presents the associations between state-level sociodemographic indicators and attitudes about COVID-19 vaccination among tweets in the United States. In multivariable associations, compared to the Republican party, the Democrat party was associated with a lower rejection rate of COVID-19 vaccination by 0.939 percentage points (95% CI –1.673 to –0.206, *P*=.01), after adjusting for the other sociodemographic factors (model 1). Notably, in model 2, after additionally adjusting for vaccine confidence, associations of the Democrat party with the rejection rate attenuated at 0.416 (95% CI –0.822 to –0.011, *P*=.04) percentage points. Indicators of vaccine hesitancy and confidence were highly correlated with each other. Consistent associations were also observed in bivariate analyses.

**Table 2:** Factors associated with hesitancy and confidence in COVID-19 vaccination across the states in the United States from bivariate and multivariable linear regressions (using state-level statistics, based on 1,812,398 tweets from all 50 US states and Washington DC).

Characteristics		Intent to accept COVID-19 vaccination				Intent to reject COVID-19 vaccination				Belief that COVID-19 vaccines are effective				Belief that COVID-19 vaccines are unsafe		
		Bivariate	Model 1^a^	Model 2^b^	Bivariate		Model 1	Model 2	Bivariate		Model 1	Model 2	Bivariate		Model 1	Model 2
**Aged ≥65 years**																
	β^c^	–.010	.048	–.102	–.004		–.029	.023	–.012		–.014	–.074	–.058		–.102	–.115
	95% CI	–0.522 to 0.502	–0.492 to 0.588	–0.355 to 0.152	–0.188 to 0.180		–0.212 to 0.154	–0.078 to 0.124	–0.145 to 0.121		–0.161 to 0.134	–0.177 to 0.029	–0.241 to 0.125		–0.285 to 0.080	–0.240 to 0.011
	*P* value	.97	.86	.43	.96		.76	.65	.85		.86	.16	.53		.27	.07
**Density (people per square mile/100)**																
	β	.059	.050	–.006	–.021		–.006	.013	.006		.002	–.017	–.030		–.032	–.030
	95% CI	–0.004 to 0.123	–0.047 to 0.146	–0.053 to 0.041	–0.044 to 0.002		–0.039 to 0.027	–0.006 to 0.032	–0.011 to 0.023		–0.025 to 0.028	–0.036 to 0.002	–0.052 to –0.008		–0.065 to 0.0005	–0.053 to –0.008
	*P* value	.07	.31	.80	.08		.72	.18	.52		.89	.08	.009		.05	.008
**Gross domestic product (GDP) per capita (ln)**																
	β	3.425	–.087	.181	–1.626		–.767	–.865	.439		.079	.217	–1.435		.232	.304
	95% CI	–0.470 to 7.320	–6.610 to 6.436	–2.779 to 3.141	–2.992 to –0.261		–2.980 to 1.446	–2.047 to 0.318	–0.597 to 1.475		–1.707 to 1.866	–1.013 to 1.448	–2.813 to –0.057		–1.969 to 2.434	–1.213 to 1.821
	*P* value	.08	.98	.91	.02		.50	.15	.40		.93	.73	.04		.84	.69
**Democrat party (Republican as the reference)**																
	β	2.217	1.894	.299	–1.132		–.939	–.416	.351		.331	–.067	–.878		–.670	–.372
	95% CI	0.267 to 4.168	–0.269 to 4.057	–0.716 to 1.313	–1.794 to –0.471		–1.673 to –0.206	–0.822 to –0.011	–0.172 to 0.874		–0.262 to 0.923	–0.489 to 0.355	–1.568 to –0.188		–1.401 to 0.060	–0.881 to 0.137
	*P* value	.03	.09	.56	.001		.01	.04	.18		.27	.75	.01		.07	.15
**Belief that COVID-19 vaccines are effective**																
	β	3.111	—^d^	1.472	–1.035		—	–.431	—		—	—	–.971		—	–.903
	95% CI	2.459 to 3.762	—	0.770 to 2.173	–1.298 to –0.771		—	–0.711 to –0.150	—		—	—	–1.252 to –0.690		—	–1.148 to –0.657
	*P* value	<.001	—	<.001	<.001		—	.003	—		—	—	<.001		—	<.001
**Belief that COVID-19 vaccines are unsafe**																
	β	–2.415	—	–1.654	.833		—	.568	–.511		—	–.594	—		—	—
	95% CI	–2.818 to –2.013	—	–2.224 to –1.085	0.672 to 0.994		—	0.340 to 0.795	–0.659 to –0.363		—	–0.755 to –0.432	—		—	—
	*P* value	<.001	—	<.001	<.001		—	<.001	<.001		—	<.001	—		—	—

^a^Multivariable linear regressions with the following covariates: political party (Democrat vs Republican as the reference) [30], population density, percentage of people aged ≥65 years, and log-transformed gross domestic product per capita.

^b^Similar to model 1 but additionally adjusted for belief in effectiveness or unsafety of COVID-19 vaccines.

^c^Determined using bivariate and multivariable linear regression analysis.

^d^—: not available.

## Discussion

### Principal Findings

This study, using social media data, monitored the trajectories of COVID-19 vaccine hesitancy and confidence in 6 high-income countries throughout the pandemic from January 2020 to June 2022. The 6 countries experienced similar evolving trends of COVID-19 vaccine hesitancy and confidence. Since the pandemic began, there has been growing hesitancy toward the COVID-19 vaccine. However, this hesitancy lessened from late 2020 to May 2021, when safety and effectiveness data for the vaccine was released. After May 2021, vaccine hesitancy started to rise again. On average, the prevalence of intent to accept COVID-19 vaccination decreased from 71% of 44,944 tweets in March 2020 to 35% of 48,167 tweets in June 2022, with fluctuations. The COVID-19 vaccine hesitancy and confidence varied by country, vaccine manufacturer, and subregion within a country. In the United States, a higher proportion of Democratic-leaning residents and higher vaccine confidence were significantly associated with lower vaccine hesitancy.

By finetuning the CT-BERT model, we conducted a cross-country social media listening study, which complements traditional public health surveillance approaches such as surveys in tackling global health challenges. During the COVID-19 pandemic, a rapidly growing body of literature has used social media listening methods to assess public attitudes toward COVID-19 vaccines. They mainly used data from Twitter to analyze public sentiment, acceptance, and topics in antivax and provax discourse toward COVID-19 vaccines [42-45]. These studies provide a solid foundation for social media listening studies on vaccines. Future studies should explore the vast potential of social media listening and how it can be integrated into existing public health surveillance systems to inform near–real-time intervention and address a wide array of global health issues.

Overall, increased COVID-19 vaccine hesitancy and decreased confidence in vaccine effectiveness and safety on Twitter were observed during 2020 and 2022. Such trends were aligned with a number of previous cross-country studies on COVID-19 vaccines [6,29,44,46], suggesting the reliability of our findings. Prior to the COVID-19 vaccine rollout, concerns and conspiracy theories surrounding COVID-19 vaccines proliferated, in part due to the rapidness of the vaccine development and the scarcity of clinical trials [47]. This might account for the rise in vaccine hesitancy during the early pandemic. Vaccine hesitancy then decreased with the release of clinical trial results in late 2020, which demonstrated vaccine effectiveness of up to 95% [48-50]. However, with the ongoing mutations of the coronavirus, vaccination hasn't been able to completely shield individuals from COVID-19 infections, nor effectively halt the virus's spread within communities. Such limitation of COVID-19 vaccines might foster distrust and cultivate conspiracy theories. When waning immunity was observed as Delta and Omicron variants started to spread in May and November 2021, respectively, vaccine hesitancy increased again [41]. On the other hand, with large-scale COVID-19 vaccination campaigns in 2021, adverse events following immunizations (AEFIs) were widely experienced, vocalized, and reported [39,51]. Google search trends on vaccine adverse events skyrocketed, indicating the rising public’s concern on vaccine safety [52]. Misinformation and rumors were also widespread, especially on social media platforms, which may exacerbate the concerns about vaccine safety and effectiveness [7,47,53,54]. Preparedness for AEFIs during mass vaccination rollout and rapid responses to misinformation could be essential to reducing the public’s vaccine hesitancy and boosting confidence, not only during the COVID-19 pandemic but also in future ones.

Despite a noticeable decline in vaccine acceptance on Twitter in 2021, real-life daily COVID-19 vaccine uptake stayed consistently high and seemed largely unaffected. This discrepancy might be partly attributed to mandatory COVID-19 vaccination policies. Under such policies, some vaccinated individuals might still harbor and express negative sentiments on social media. It may also stem from gaps between the general population and Twitter users. Vocal antivaccine groups on Twitter might have formed a tight-knit circle, continuously congregating and reinforcing their messages, which could be a significant factor in the perceived decline in vaccine acceptance observed in our data set [55].

The differential prevalence of vaccine hesitancy and confidence by country and manufacturer were also aligned with previous studies [2,4,7,56,57]. Since AstraZeneca and Johnson & Johnson vaccination paused after related AEFI reports [39,58,59], vaccine acceptance rates for these 2 vaccines were lower in most of, but not all, the 6 high-income countries. The UK Twitter users held a higher acceptance rate of the AstraZeneca vaccine manufactured based in the United Kingdom, suggesting that the location of the manufacturer might play an important role in vaccine hesitancy. A survey also showed that the French population reported the lowest hesitancy for vaccines manufactured in the European Union, but higher hesitancy for vaccines manufactured in the United States or China [56].

We found that in the United States, Democrat-leaning states were estimated to be significantly lower in vaccine hesitancy, which was consistent with a previous survey indicating that Republicans exhibited more negative sentiments toward COVID-19 vaccination than Democrats [6]. The party gap in vaccine hesitancy further led to the excess mortality gap between Republicans and Democrats following the deployment of COVID-19 vaccination [60]. Differential exposure to media channels and public figures may explain the observed gap in vaccine hesitancy between self-identified Democrats and Republicans. Democrat-leaning states had a higher percentage of COVID-19 cases in early pandemic [61], and also due to different sources of information, Democrats perceived the COVID-19 threat to be greater than Republicans [6]. Compared to the Democrats, the trust in the media decreased significantly during the pandemic among the Republicans [6], whereas misinformation on COVID-19 vaccinations may be more likely to spread in people who don’t trust the information source (ie, Republicans). Our study highlights the necessity to bridge the vaccine confidence gap and prevent death due to political affiliation, particularly addressing the rising trends of vaccine hesitancy among Republicans.

Our study is subject to several limitations. Twitter is more commonly used by the younger generations [62], literate people, and those with access to the internet. Therefore, discourse on Twitter might not reflect the broader population, and our ecological analysis might be biased. However, by analyzing all English-language tweets containing COVID-19 vaccine–related keywords with a state-of-the-art deep learning model, our results are valid and robust enough to represent the opinion of Twitter users in the 6 high-income countries, and are aligned with previous survey studies [8,46]. Furthermore, our correlation analysis was also restricted to state-level statistics in the United States. Examining other factors associated with COVID-19 vaccination using a larger sample size should be considered in further studies. Finally, future analyses should be conducted to explain the trajectories in COVID-19 vaccine hesitancy and confidence in the 6 countries.

Our study also has several notable strengths. To begin with, we continuously monitored vaccine hesitancy and confidence throughout the entire pandemic in 6 countries, which is often infeasible for survey studies. This longitudinal analysis enhances our understanding of evolving vaccine attitudes and strengthens the preparedness for future pandemics. Second, compared to survey studies, social media data provides a cost-efficient approach to tracking the trajectory of vaccine hesitancy and confidence, facilitating near–real-time public health interventions. Third, this study provides a pathway to monitor the subsequent “infodemics” of misinformation, rumor, and distrust using advanced machine learning approaches. While social media is a predominant place for the breeding of the “infodemic” that poses a challenge to public health response during the pandemic, studies that target the infodemic and its linkages to factors such as sociodemographic indicators are limited. Future studies may leverage more in-depth analyses using social media data for detecting, tracking, and addressing the “infodemic.” Overall, this study finetuned state-of-the-art deep learning models using a task-specific data set as a rapid and effective approach to monitoring vaccine hesitancy and confidence, which provides a more reliable estimation of vaccine hesitancy and confidence.

### Conclusions

With an advanced deep learning model and large-scale social media data, this study tracked the trajectory of COVID-19 vaccine acceptance and confidence on Twitter in 6 high-income countries. We highlighted the similarity in the temporal trends in hesitancy and confidence across countries, which were influenced by the development of vaccines and the evolution of viruses throughout the pandemic. This study also revealed the discrepancy across regions and vaccine manufacturers and that the spatial variation may be associated with political ideology. This surveillance study highlights the importance of deep learning-based social media monitoring to detect emerging trends to inform timely interventions and provide insight not yet covered in previous surveys. Future studies should leverage deep learning models as a rapid and effective approach to monitor public hesitancy toward varying kinds of vaccines in real time, with data from multiple social media platforms.

## Appendix

Multimedia Appendix 1 Details of methods, Tables S1-S5, and Figures S1-S4.

## Abbreviations

AEFI: adverse events following immunization
BERT: Bidirectional Encoder Representations from Transformer
CT-BERT: COVID-Twitter-Bidirectional Encoder Representations from Transformer
NLP: natural language processing

## Data Availability

Results and Python code for data analysis can be available on request by contacting the corresponding author (ZH). However, the original tweet data cannot be shared according to Twitter's Terms of Service.
