# Supplementary material for: Deep Learning Analysis of COVID-19 Vaccine Hesitancy and Confidence Expressed on Twitter in 6 High-Income Countries: Longitudinal Observational Study
Source: J Med Internet Res. 2023 Nov 6;25:e49753. doi: 10.2196/49753 (PMC10629504; doi:10.2196/49753)

**Deep learning analysis details**

We employed a *hierarchical* classification strategy to analysis COVID-19 vaccine tweets.

First, we train a deep learning model based on CT-BERT to distinguish if a tweet was likely to be sent by human or not. For this purpose, all 8,073 manually-annotated tweets were involved, including 4,184 tweets annotated likely to be human-generated and 3,889 tweets annotated as not likely to be human-generated. From this dataset, 6,546 tweets constituted the training set, 727 were allocated to the development set, and 800 were reserved for the test set. Upon evaluation using the held-out test set, our model achieved an F1-score of 0.86. Subsequently, we deployed the model to classify the vast collection of tweets from six countries, totaling 5,257,385 tweets. Of these, 3,348,746 tweets were assessed as likely to be human-generated.

Second, we train multiple deep learning models based on CT-BERT to categorize human-generated tweets (n=3,348,746) based on their attitudes towards our four predefined criteria: accept, reject, effective, and not safe. In this phase, we solely focused on tweets manually tagged as “likely to be human-generated” (n=4,184) to train the model; Specifically, 2,129 tweets expressed an “Intent to accept COVID-19 vaccination,” 543 showed an “Intent to reject COVID-19 vaccination,” 749 conveyed a “Belief that the COVID-19 vaccine is effective,” and 334 indicated a “Belief that the COVID-19 vaccine is not safe.” Using the models developed in this phase, we categorized each human-generated tweet into one, multiple, or none of the predefined categories. Of the manually annotated set, 3,400 tweets were used for training, 378 for validation, and 406 for testing. In the held-out test set, our model displayed F1-scores between 0.7 and 0.86. We then applied our trained model to the entire collection of tweets identified as likely human-generated (n=3,348,746) and classified them into the four categories.

Details on the definitions of our categories and model performance for each category can be found in Table S1 in Multimedia Appendix 1. Hyperparameters utilized for training the models and the number of tweets in each category can be found in Table S2 in Multimedia Appendix 1.

**Table S1.** Annotation categories and performance of deep learning models in annotating COVID-19 vaccine-related tweets.

Layer 1.

| Annotation category | Definition | Model performance | |
| --- | --- | --- | --- |
|  |  | **F_1_-score** | **Precision** |
| (Likely to be) Human generated | Twitter® posts were considered likely to be human generated. (Non-human-generated tweets are normally sent by bots, new agencies, and governments) | 0.86 | 0.89 |

Layer 2.

| **Annotation category** | **Definition** | **Model performance** | |
| --- | --- | --- | --- |
|  |  | **F_1_-score** | **Precision** |
| **Vaccine hesitancy** | | | |
| **Intent to *accept* COVID-19 vaccination** | Twitter® posts indicating that they will accept, support or be willing to get COVID-19 vaccination. | 0.86 | 0.88 |
| **Intent to *reject* COVID-19 vaccination** | Twitter® posts indicating that they will reject, do not support, or be unwilling to get COVID-19 vaccination. | 0.75 | 0.78 |
| **Vaccine confidence** | | | |
| **Belief that COVID-19 vaccine is *effective*** | Twitter® users had confidence in the effectiveness of the COVID-19 vaccine, believing that it is effective. | 0.84 | 0.83 |
| **Belief that COVID-19 vaccine is *not safe*** | Twitter® users had lack of confidence in the safety of the COVID-19 vaccine, believing it was not safe. | 0.70 | 0.66 |

**Table S2**. The number of tweets in each category in the finetuning dataset and model finetuning hyperparameters.

Layer 1.

| **Annotation category** | **Hyperparameters** | **Number of tweets** | |
| --- | --- | --- | --- |
|  |  | **Training set (90%) and validation set (10%)**  **(n=7273 in total)** | **Test set**  **(n=800)** |
| **Human generated** | max length=96, batch size=32, optimizer: AdamW, lr=1.7e-5, weight decay=1e-4, epoch=3. | 3778 | 406 |

Layer 2.

| **Annotation category** | **Hyperparameters** | **Number of tweets** | |
| --- | --- | --- | --- |
|  |  | **Training set (90%) and validation set (10%)**  **(n=3778 in total)** | **Test set**  **(n=406)** |
| **Vaccine hesitancy** | | | |
| **Intent to *accept* COVID-19 vaccination** | max length=96, batch size=32, optimizer: AdamW, learning rate=1.5e-5, weight decay=1e-4, epoch=4 | 1919 | 210 |
| **Intent to *reject* COVID-19 vaccination** | max length=96, batch size=32, optimizer: AdamW, learning rate=1.7e-5, weight decay=1e-4, epoch=3 | 492 | 51 |
| **Vaccine confidence** | | | |
| **Belief that COVID-19 vaccine is *effective*** | max length=96, batch size=32, optimizer: AdamW, learning rate=3.1e-5, weight decay=5e-4, epoch=6; | 679 | 70 |
| **Belief that COVID-19 vaccine is *not safe*** | max length=96, batch size=32, optimizer: AdamW, learning rate=3.1e-5, weight decay=1e-4, epoch=5; | 306 | 28 |

**Table S3**. Keywords for identifying vaccine manufacturers.

| Manufacturer | Keywords (ignore case) | Number of tweets  (3,348,746 in total) |
| --- | --- | --- |
| Johnson & Johnson | johnson and johnson, johnsonandjohnson, Johnson & Johnson, Johnson&Johnson, J&J, Janssen, Ad26.COV2.S, JNJ-78436735, Ad26COVS1, VAC31518, JNJ Covid vaccine, jnj vaccine, JnJ #vaccine | 4,148 (0.1%) |
| Moderna | Moderna, Spikevax, mRNA-1273, nct04470427, CX-024414, TAK-919, mRNA-1273.211 | 46,529 (1.4%) |
| AstraZeneca | AstraZeneca, Astra-Zeneca, Vaxzevria, azd1222, ChAdOx1 nCoV-19, ChAdOx1-S, AZD2816, NCT04516746 | 22,006 (0.7%) |
| Pfizer | Pfizer, biontech, fosun, BNT162b2, Comirnaty, BNT162b1, NCT04368728 | 110,679 (3.3%) |

**Table S4.** State-level sociodemographic characteristics in the United States.

|  | Political party^a^ | Population density^c^ | Percent 65 years and older^d^ | GDP per capita^e^ |
| --- | --- | --- | --- | --- |
|  | 2020 | People per square mile, 2020 | %, 2019 | $, 2021 |
| District of Columbia | Democratic | 11280.7 | 12.4 | 174500 |
| Maryland | Democratic | 636.1 | 15.9 | 51724 |
| New Jersey | Democratic | 1263.0 | 16.6 | 56477 |
| Delaware | Democratic | 508.0 | 19.5 | 69667 |
| Connecticut | Democratic | 744.7 | 17.6 | 64833 |
| Rhode Island | Democratic | 1,061.4 | 17.7 | 45000 |
| Massachusetts | Democratic | 901.2 | 17.0 | 58108 |
| California | Democratic | 253.7 | 14.8 | 51914 |
| Illinois | Democratic | 230.8 | 16.1 | 50328 |
| Ohio | Republican | 288.8 | 17.5 | 42035 |
| Pennsylvania | Democratic | 290.6 | 18.7 | 45323 |
| New York | Democratic | 428.7 | 16.9 | 57423 |
| Virginia | Democratic | 218.6 | 15.9 | 53463 |
| North Carolina | Republican | 214.7 | 16.7 | 42884 |
| Florida | Republican | 401.4 | 20.9 | 40106 |
| Hawaii | Democratic | 226.6 | 19.0 | 49214 |
| Washington | Democratic | 115.9 | 15.9 | 52403 |
| Texas | Republican | 111.6 | 12.9 | 45940 |
| Louisiana | Republican | 107.8 | 16.0 | 47467 |
| Georgia | Democratic | 185.6 | 14.3 | 41711 |
| South Carolina | Republican | 170.2 | 18.2 | 35717 |
| Tennessee | Republican | 167.6 | 16.7 | 39730 |
| Kentucky | Republican | 114.1 | 16.9 | 37535 |
| Indiana | Republican | 189.4 | 16.1 | 41169 |
| Michigan | Democratic | 178 | 17.7 | 37616 |
| Wisconsin | Democratic | 108.8 | 17.5 | 44105 |
| New Hampshire | Democratic | 153.8 | 18.6 | 47385 |
| Oregon | Democratic | 44.1 | 18.2 | 44447 |
| Nevada | Democratic | 28.3 | 16.2 | 47222 |
| Arizona | Democratic | 62.9 | 18.0 | 40828 |
| Utah | Republican | 39.7 | 11.4 | 41750 |
| Colorado | Democratic | 55.7 | 14.7 | 51940 |
| Nebraska | Republican | 25.5 | 16.1 | 49778 |
| Kansas | Republican | 35.9 | 16.4 | 44310 |
| Oklahoma | Republican | 57.7 | 16.1 | 42237 |
| Minnesota | Democratic | 71.7 | 16.3 | 50396 |
| Iowa | Republican | 57.1 | 17.5 | 49067 |
| Missouri | Republican | 89.5 | 17.2 | 41117 |
| Arkansas | Republican | 57.9 | 17.4 | 36483 |
| Mississippi | Republican | 63.1 | 16.4 | 32967 |
| Alabama | Republican | 99.2 | 17.4 | 36333 |
| West Virginia | Republican | 74.6 | 20.5 | 35053 |
| Vermont | Democratic | 69.8 | 20.1 | 44000 |
| Maine | Democratic | 44.2 | 21.3 | 40923 |
| Idaho | Republican | 22.3 | 16.2 | 34250 |
| Montana | Republican | 7.4 | 19.5 | 37200 |
| Wyoming | Republican | 5.9 | 17.1 | 63667 |
| North Dakota | Republican | 11.3 | 15.8 | 47714 |
| South Dakota | Republican | 11.7 | 17.4 | 49875 |
| Alaska | Republican | 1.3 | 12.4 | 65143 |
| New Mexico | Democratic | 17.5 | 18.0 | 35952 |

Note:

^a^data resource: <https://commons.wikimedia.org/wiki/File:ElectoralCollege2020.svg#mw-jump-to-license>.

^b^data resource: <https://www.census.gov/library/visualizations/interactive/race-and-ethnicity-in-the-united-state-2010-and-2020-census.html>

^c^data resource: <https://www.census.gov/library/visualizations/interactive/2020-population-and-housing-state-data.html>

^d^data resource: <https://www.census.gov/library/visualizations/interactive/population-65-and-older-2019.html>

^e^data resource: <https://zh.wikipedia.org/wiki/%E7%BE%8E%E5%9C%8B%E5%90%84%E5%B7%9E%E4%BA%BA%E5%9D%87%E7%94%9F%E7%94%A2%E7%B8%BD%E9%A1%8D%E5%88%97%E8%A1%A8>

**Table S5.** Descriptive statistics of COVID-19 vaccine-related tweets sent by humans in 6 countries.

| Subregion | Total number of users | Total number of tweet | Intent to accept COVID-19 vaccination | Intent to reject COVID-19 vaccination | Belief that COVID-19 vaccine is effective | Belief that COVID-19 vaccine is not safe |
| --- | --- | --- | --- | --- | --- | --- |
| *United States* | | | | | | |
| California | 109002 | 291032 | 52.5% | 4.7% | 10.2% | 10.1% |
| New York | 64209 | 176702 | 52.8% | 4.6% | 9.7% | 9.1% |
| Texas | 52975 | 124541 | 48.6% | 6.6% | 8.8% | 11.2% |
| Florida | 45019 | 121996 | 44.5% | 7.2% | 8.4% | 11.3% |
| Pennsylvania | 28781 | 73563 | 51.8% | 5.2% | 9.7% | 9.6% |
| Illinois | 26855 | 67037 | 54.0% | 4.4% | 10.2% | 9.2% |
| Ohio | 24554 | 59313 | 50.4% | 6.6% | 10.1% | 10.7% |
| District of Columbia | 18084 | 53627 | 57.8% | 3.5% | 10.8% | 7.3% |
| Georgia | 21927 | 52830 | 46.3% | 7.9% | 9.1% | 11.2% |
| Michigan | 19035 | 48152 | 50.7% | 6.3% | 9.8% | 10.8% |
| Massachusetts | 18010 | 47894 | 58.9% | 3.4% | 11.5% | 7.6% |
| New Jersey | 17420 | 44617 | 49.6% | 6.0% | 9.2% | 10.0% |
| Washington | 16721 | 43658 | 56.2% | 3.6% | 11.1% | 9.1% |
| Arizona | 15895 | 43175 | 47.4% | 7.0% | 9.3% | 11.8% |
| Virginia | 16440 | 42766 | 52.8% | 5.3% | 10.8% | 9.6% |
| North Carolina | 15198 | 37273 | 52.4% | 5.5% | 10.4% | 9.5% |
| Maryland | 13512 | 35874 | 53.7% | 4.6% | 11.1% | 9.1% |
| Minnesota | 13129 | 34882 | 54.9% | 4.4% | 10.9% | 9.4% |
| Tennessee | 13560 | 33136 | 48.5% | 6.6% | 9.9% | 10.2% |
| Oregon | 12199 | 32157 | 54.5% | 4.6% | 10.8% | 9.9% |
| Colorado | 11686 | 30002 | 54.3% | 4.3% | 10.6% | 9.6% |
| Missouri | 9914 | 25945 | 53.6% | 5.1% | 10.6% | 9.6% |
| Wisconsin | 9708 | 25339 | 52.7% | 4.9% | 10.8% | 9.6% |
| Nevada | 8426 | 22045 | 45.4% | 7.4% | 9.4% | 12.7% |
| Indiana | 9189 | 21462 | 53.3% | 5.6% | 11.0% | 9.6% |
| Connecticut | 7769 | 21357 | 54.7% | 4.3% | 10.9% | 9.4% |
| South Carolina | 7597 | 19122 | 46.0% | 7.7% | 9.0% | 11.4% |
| Louisiana | 6763 | 16107 | 45.6% | 8.3% | 8.9% | 11.6% |
| Alabama | 6568 | 15980 | 50.8% | 6.3% | 9.8% | 9.9% |
| Kentucky | 6516 | 15634 | 53.5% | 5.6% | 11.7% | 10.0% |
| Oklahoma | 5263 | 12319 | 50.5% | 6.8% | 10.1% | 10.8% |
| Utah | 4727 | 11248 | 54.6% | 4.2% | 11.2% | 10.3% |
| Iowa | 4702 | 11188 | 55.6% | 4.2% | 11.1% | 8.4% |
| Kansas | 4372 | 10546 | 54.4% | 5.0% | 12.2% | 9.4% |
| Arkansas | 4113 | 9909 | 52.4% | 5.7% | 9.8% | 10.4% |
| Hawaii | 2918 | 7988 | 47.3% | 5.3% | 9.6% | 11.3% |
| Idaho | 2749 | 7658 | 51.9% | 5.6% | 10.0% | 10.5% |
| New Hampshire | 2912 | 7636 | 53.0% | 5.2% | 10.4% | 9.5% |
| Nebraska | 3021 | 7379 | 53.3% | 5.4% | 11.6% | 9.0% |
| New Mexico | 2851 | 7315 | 53.2% | 5.0% | 11.2% | 10.4% |
| West Virginia | 2971 | 6989 | 53.7% | 5.8% | 10.9% | 10.0% |
| Maine | 2233 | 5775 | 55.4% | 3.8% | 10.6% | 8.7% |
| Mississippi | 2195 | 4871 | 47.2% | 7.6% | 9.4% | 11.7% |
| Alaska | 1593 | 4767 | 49.8% | 6.0% | 10.1% | 11.5% |
| Rhode Island | 1841 | 4724 | 57.6% | 4.2% | 12.0% | 8.5% |
| Montana | 1202 | 3307 | 53.0% | 5.2% | 9.4% | 9.5% |
| Vermont | 982 | 2903 | 59.9% | 3.2% | 11.2% | 7.6% |
| South Dakota | 1045 | 2789 | 48.9% | 5.7% | 10.6% | 10.6% |
| Delaware | 1116 | 2678 | 51.0% | 5.2% | 11.3% | 9.7% |
| North Dakota | 756 | 1696 | 52.1% | 4.9% | 10.3% | 12.1% |
| Wyoming | 550 | 1495 | 45.5% | 8.7% | 7.8% | 14.2% |
|  |  |  |  |  |  |  |
| *United Kingdom* | | | | | | |
| England | 142456 | 408135 | 59.6% | 3.1% | 10.5% | 8.6% |
| Scotland | 17015 | 43940 | 59.8% | 3.6% | 10.4% | 8.0% |
| Wales | 7593 | 21502 | 60.2% | 3.1% | 10.0% | 8.2% |
| Northern Ireland | 3339 | 8881 | 59.7% | 3.3% | 11.0% | 9.4% |
| *Australia* | | | | | | |
| Victoria | 10672 | 37899 | 54.4% | 2.8% | 10.7% | 9.3% |
| New South Wales | 9392 | 34372 | 53.7% | 2.9% | 10.3% | 9.2% |
| Queensland | 4439 | 14943 | 50.5% | 4.0% | 9.5% | 11.3% |
| Western Australia | 2490 | 8576 | 50.8% | 3.9% | 9.7% | 11.0% |
| South Australia | 1759 | 5923 | 53.7% | 2.7% | 10.2% | 10.1% |
| Australian Capital Territory | 716 | 2649 | 64.3% | 1.0% | 10.8% | 4.3% |
| Tasmania | 439 | 1390 | 53.3% | 2.1% | 7.9% | 8.1% |
|  |  |  |  |  |  |  |
| *Canada* | | | | | | |
| Ontario | 34236 | 105916 | 57.7% | 3.2% | 11.0% | 8.4% |
| British Columbia | 11283 | 36726 | 55.7% | 3.6% | 11.7% | 9.4% |
| Alberta | 10044 | 31737 | 51.9% | 4.3% | 10.7% | 10.7% |
| Manitoba | 2408 | 7408 | 57.6% | 3.3% | 11.9% | 9.0% |
| Nova Scotia | 2243 | 5885 | 59.2% | 2.9% | 12.3% | 8.5% |
| Saskatchewan | 1756 | 4884 | 56.5% | 3.5% | 11.4% | 8.5% |
| Quebec | 1546 | 4765 | 54.2% | 2.7% | 9.4% | 9.8% |
| New Brunswick | 867 | 2551 | 58.7% | 3.7% | 10.5% | 10.1% |
| Prince Edward Island | 186 | 526 | 60.8% | 3.8% | 12.5% | 9.4% |
| Newfoundland and Labrador | 139 | 277 | 69.3% | 1.5% | 15.7% | 7.9% |
|  |  |  |  |  |  |  |
| *New Zealand* | | | | | | |
| Auckland | 1435 | 5072 | 61.0% | 2.3% | 11.8% | 8.0% |
| Wellington | 844 | 2539 | 64.1% | 2.0% | 11.5% | 7.7% |
| Canterbury | 345 | 943 | 56.1% | 2.6% | 11.9% | 10.2% |
| Otago | 121 | 397 | 70.3% | 0.9% | 9.8% | 5.7% |
|  |  |  |  |  |  |  |
| *Ireland* | | | | | | |
| County Dublin | 3190 | 9570 | 64.4% | 2.1% | 10.8% | 6.0% |
| County Cork | 1133 | 2904 | 65.3% | 2.9% | 13.2% | 6.7% |
| County Kildare | 240 | 851 | 59.4% | 3.1% | 11.7% | 7.8% |
| County Limerick | 300 | 743 | 62.3% | 2.2% | 11.5% | 8.9% |
| County Waterford | 206 | 453 | 64.2% | 1.0% | 12.4% | 6.0% |
| County Kerry | 100 | 352 | 55.2% | 4.3% | 12.9% | 11.4% |

Note: only subregions with tweets from not less than 100 users were included.

**Figure S1.** Number of COVID-19 vaccine-related tweets and users monthly in each country.


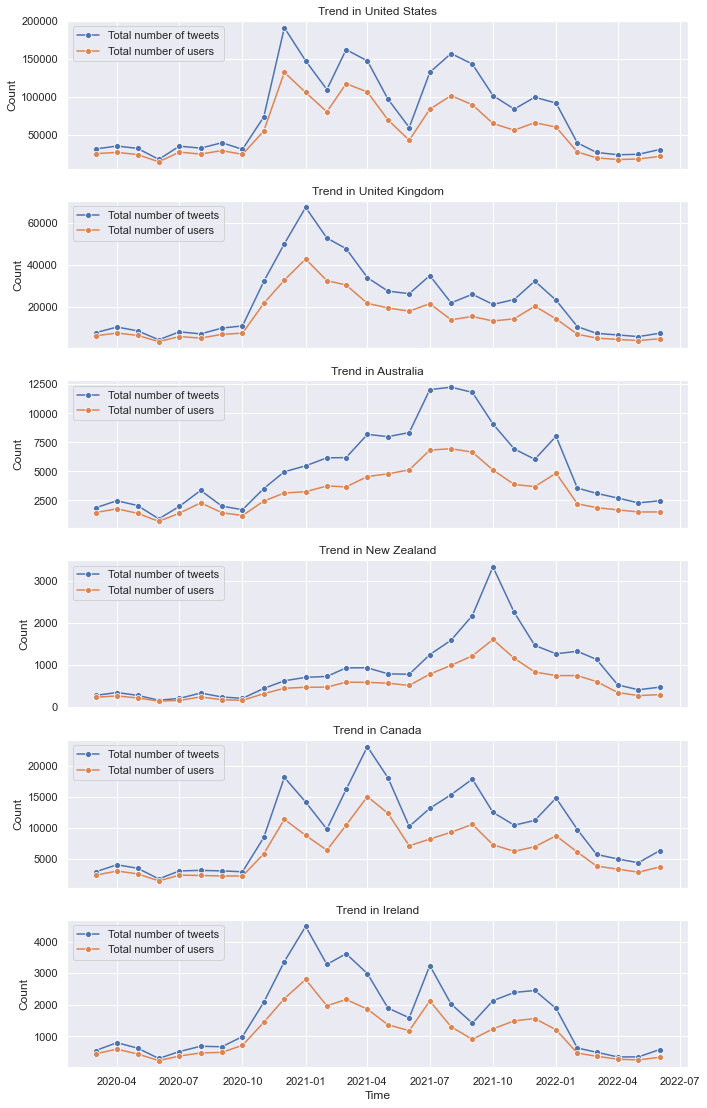


**Figure S2.** Monthly COVID-19 vaccination trends in six countries.

- Trends in Canada, Ireland, and United States are shown by vaccine brand, whereas in Australia, New Zealand, and United Kingdom, only the overall trends are shown, due to a lack of data availability. Data were collected from <https://ourworldindata.org/>.


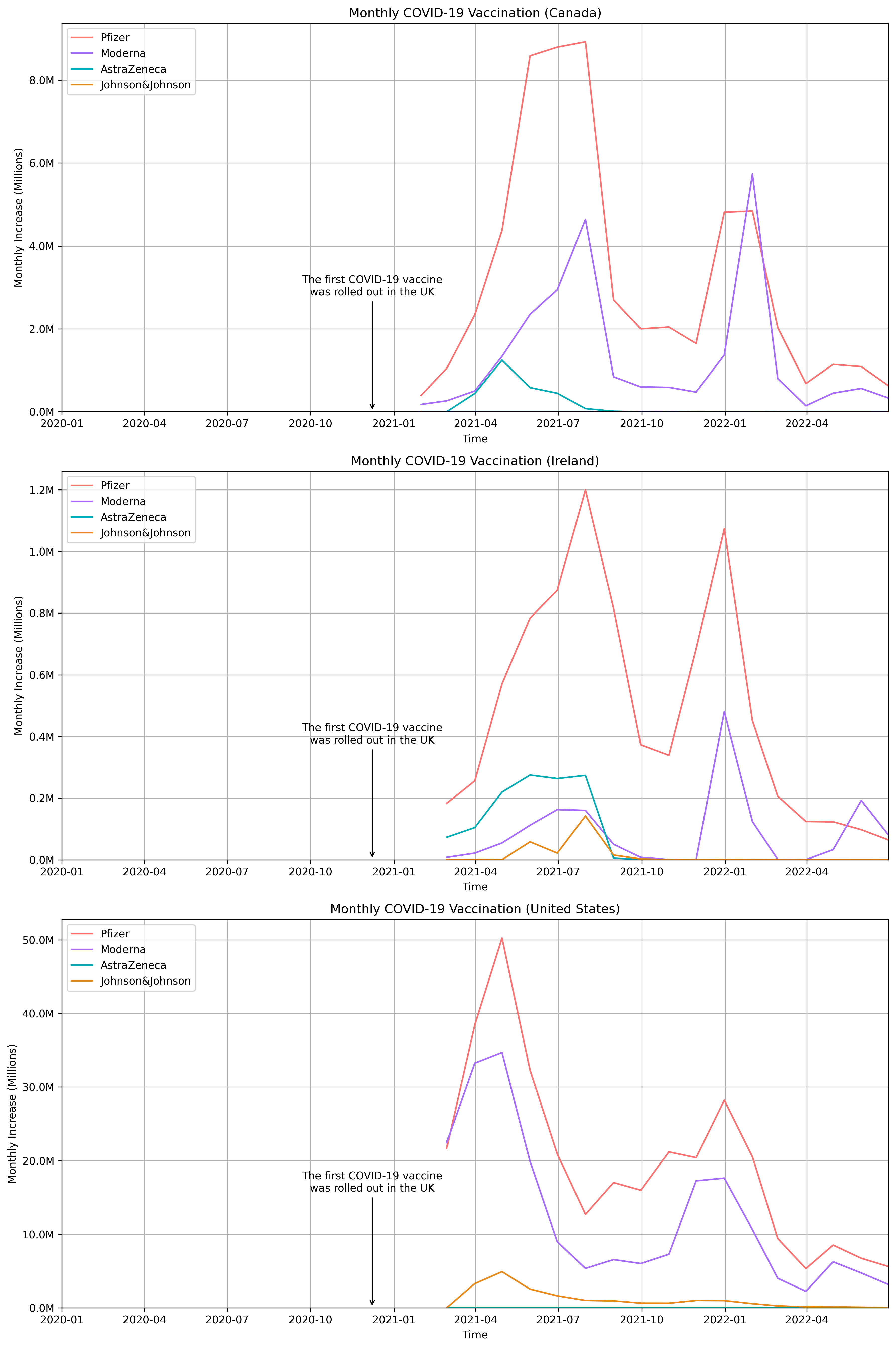


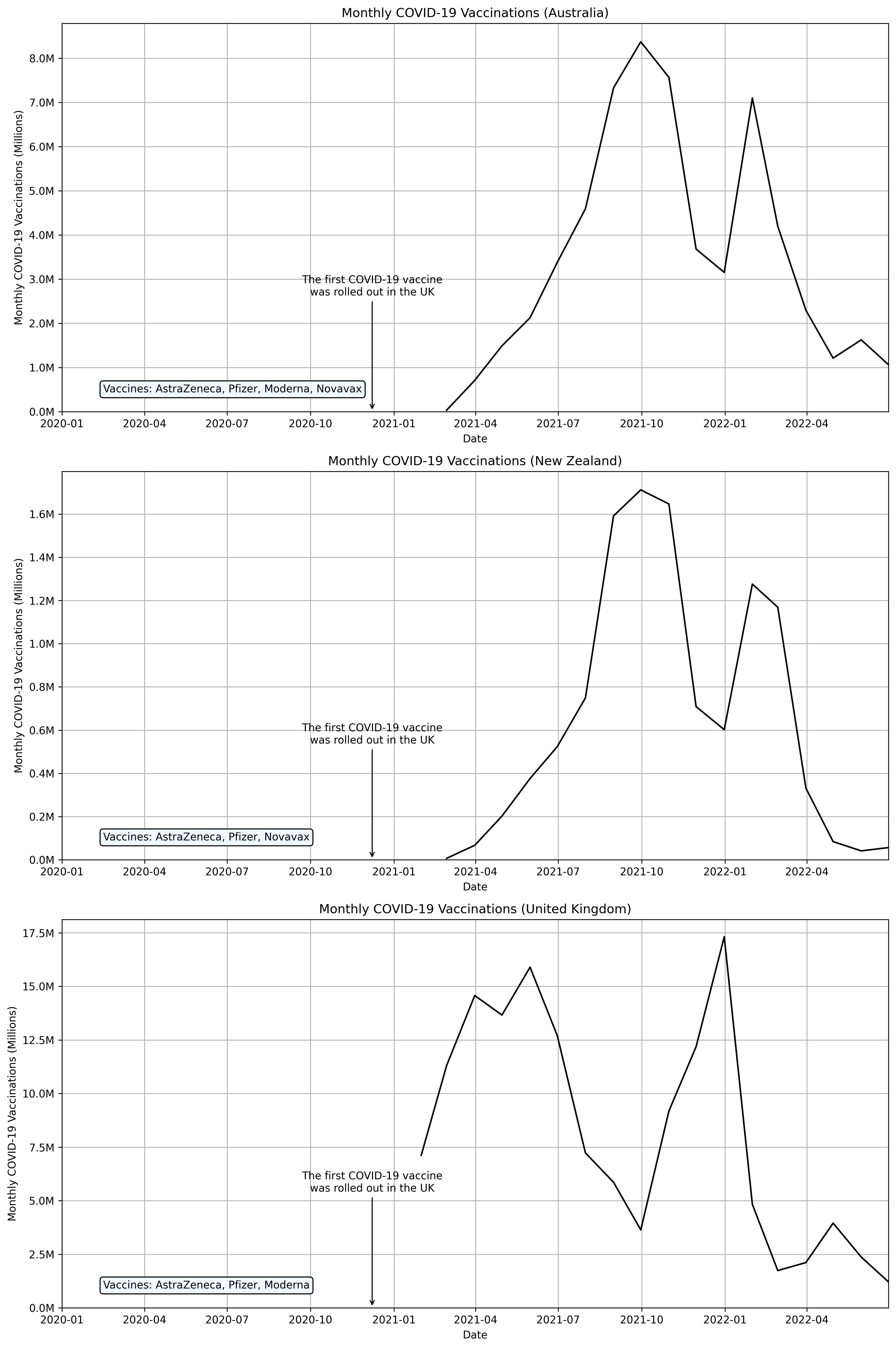


**Figure S3.** Time lag correlation.

**Figure S3A.** Time lag correlation between COVID-19 vaccine acceptance and belief in effectiveness in six countries.


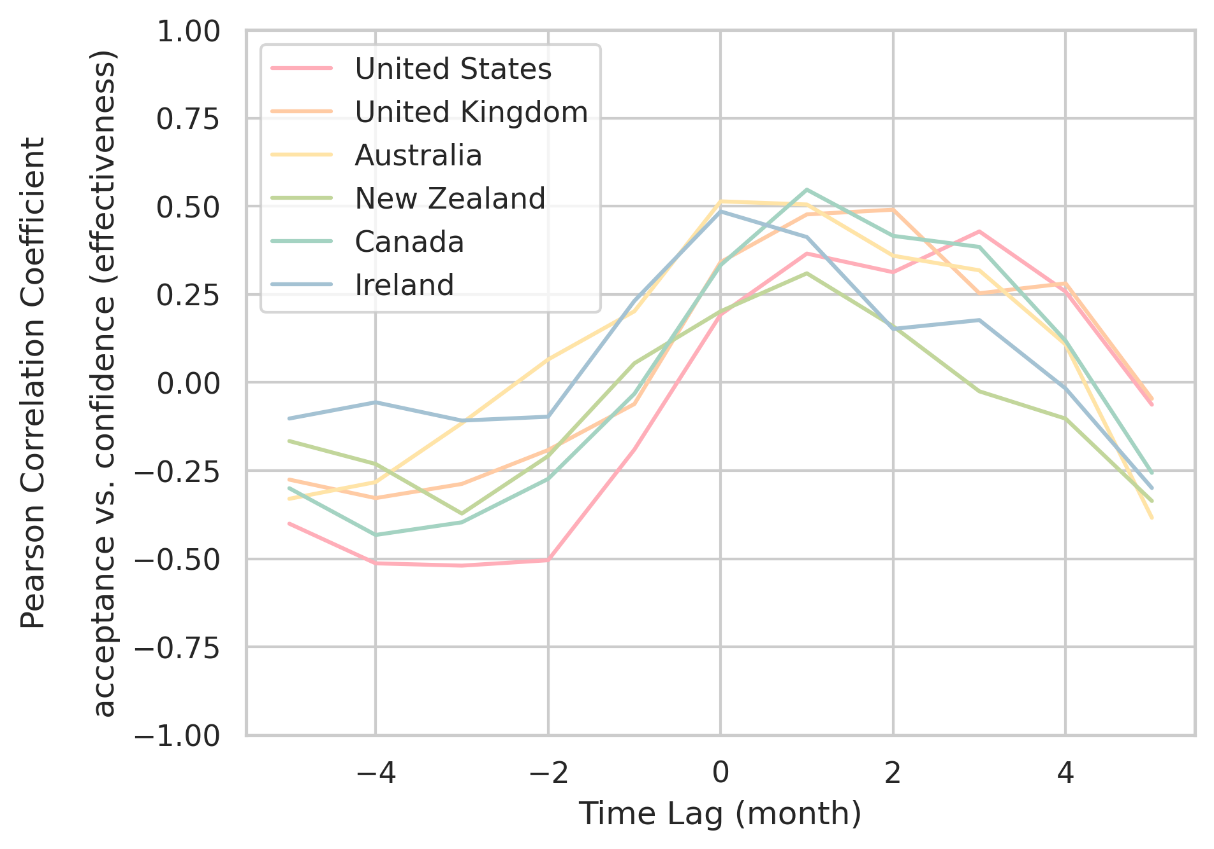


**Figure S3B.** Time lag correlation between COVID-19 vaccine acceptance and rejection in six countries.


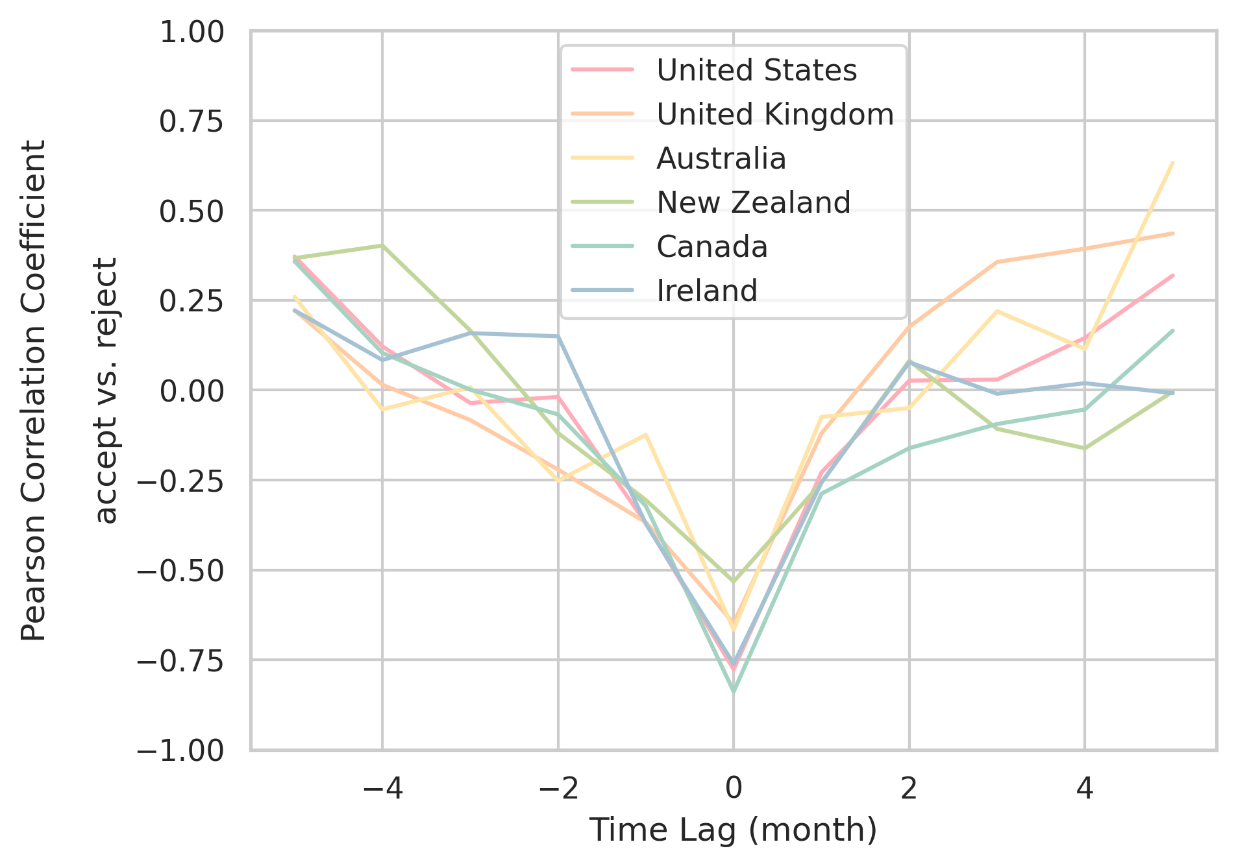


**Figure S4.** Joinpoint trend analysis.

1. Intent to accept COVID-19 vaccination


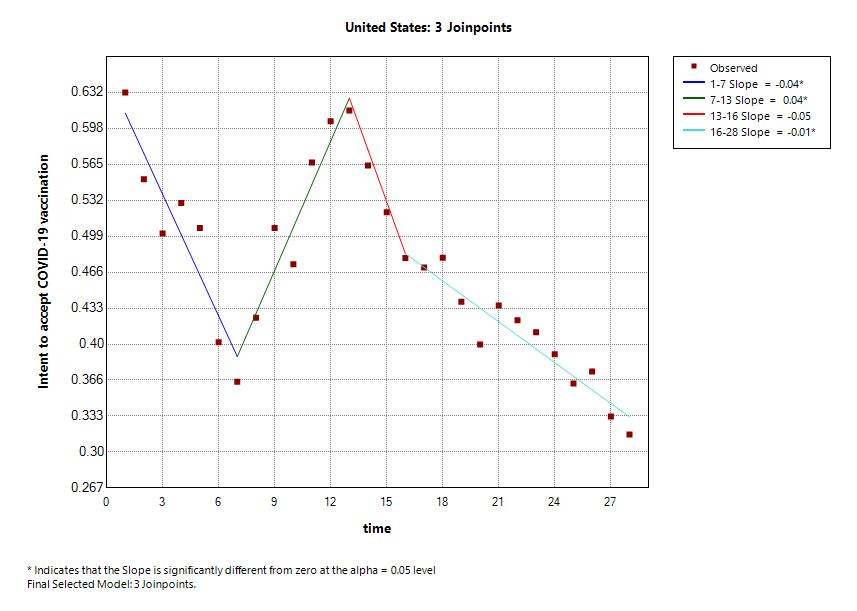


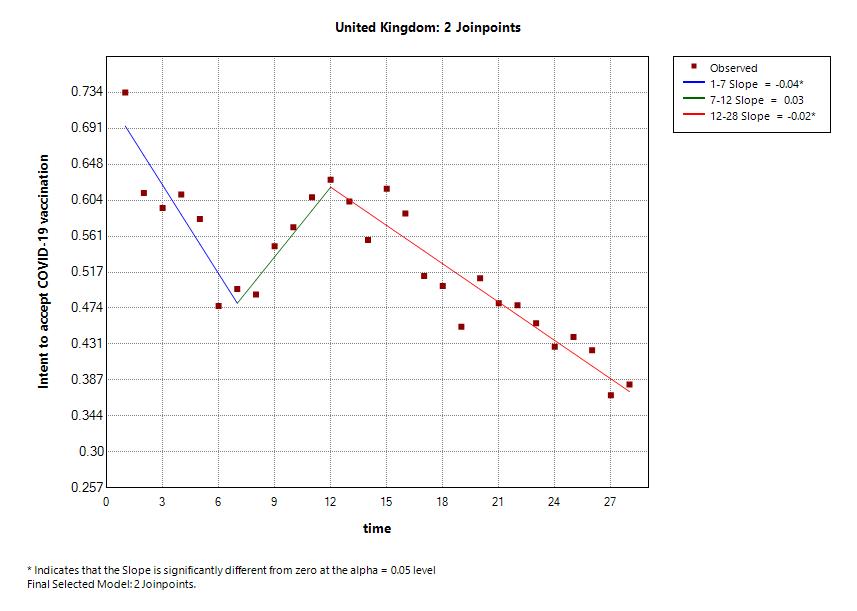


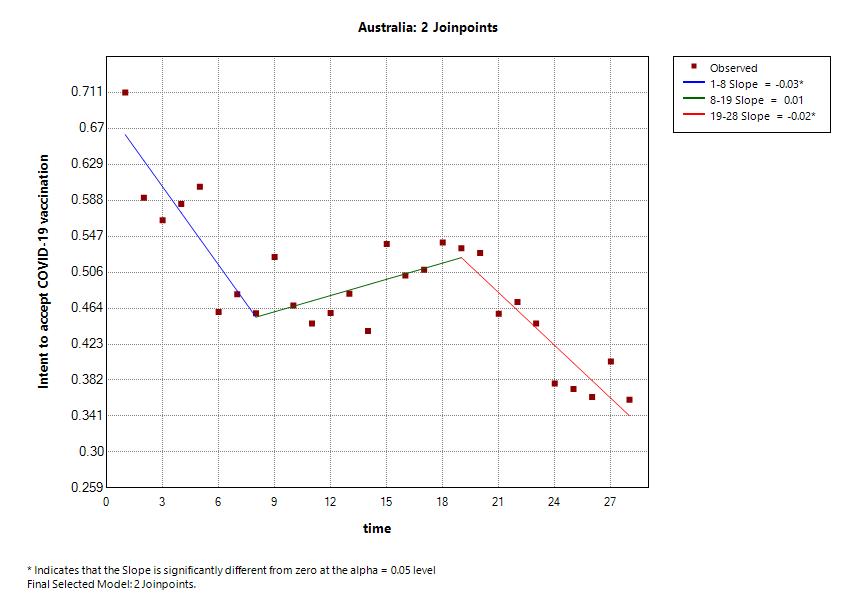


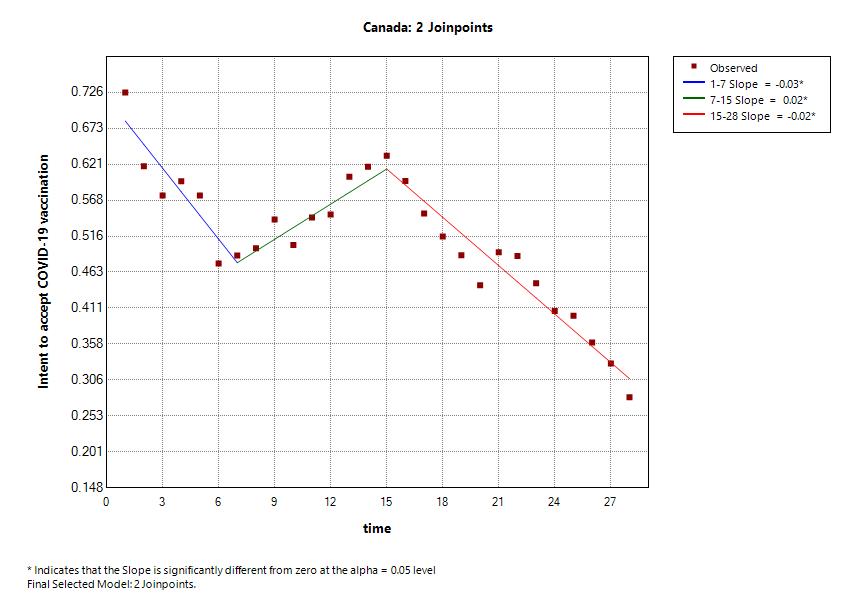


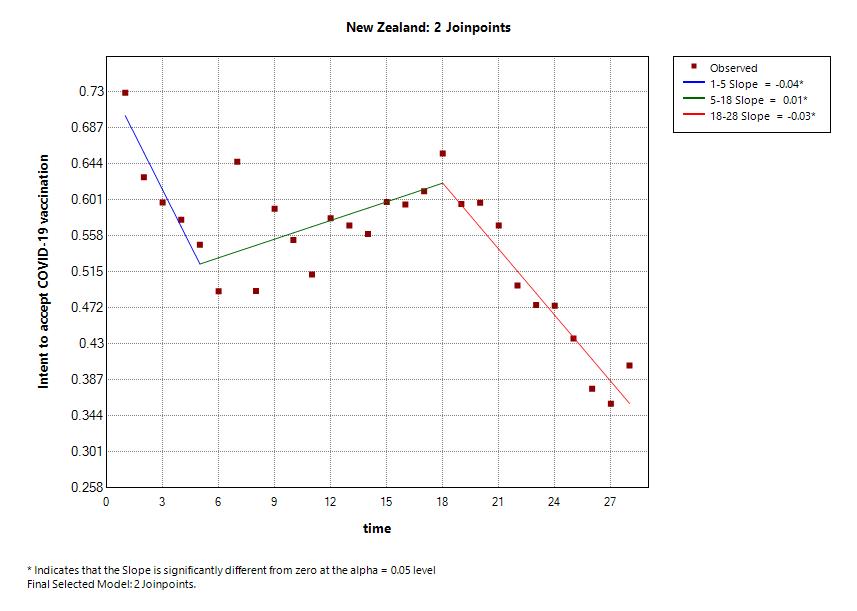


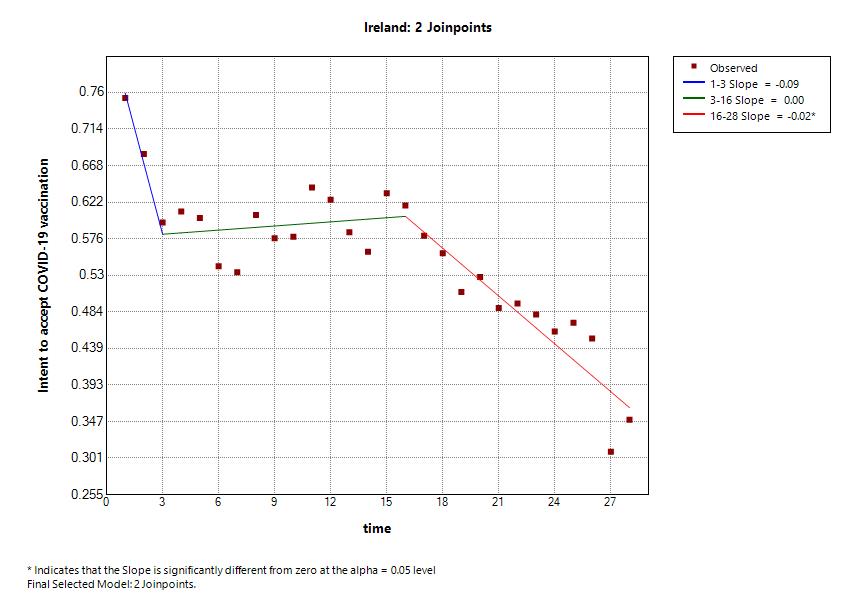


1. Intent to reject COVID-19 vaccination


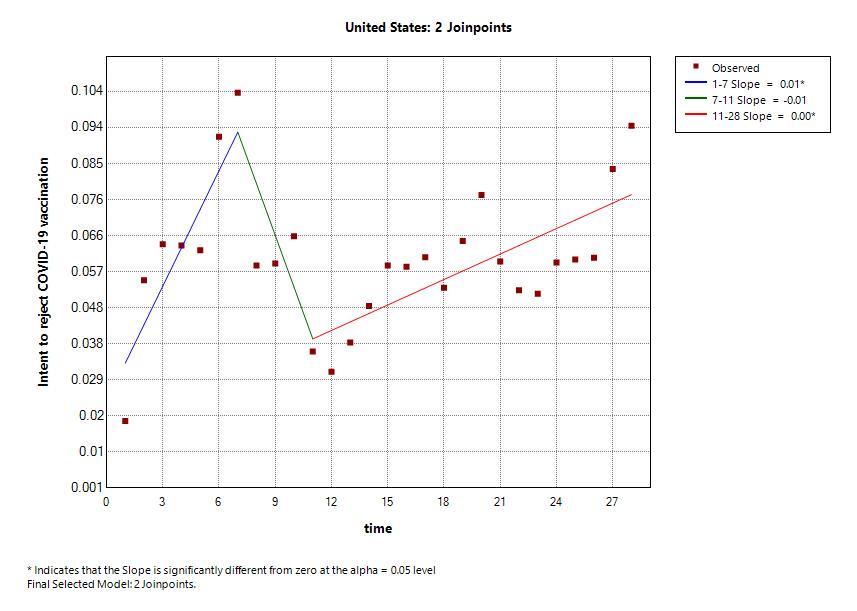


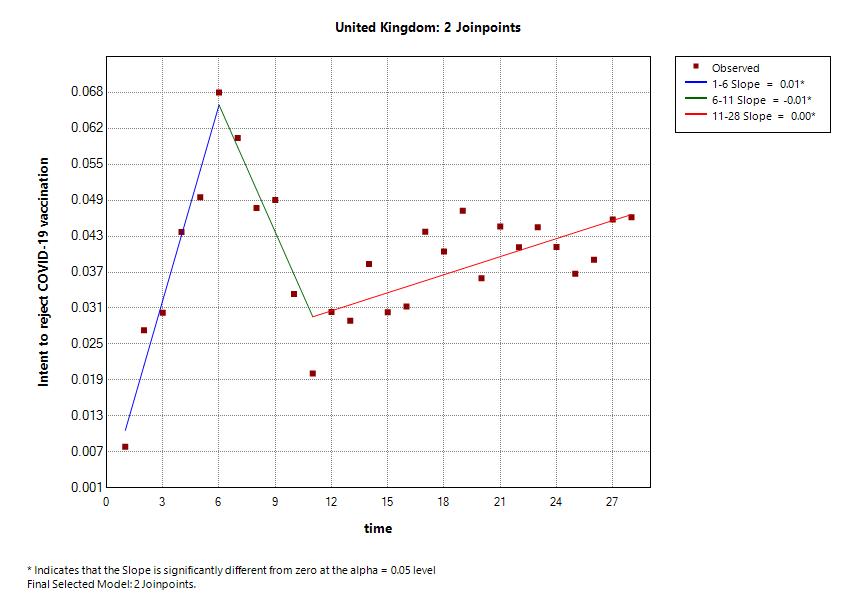

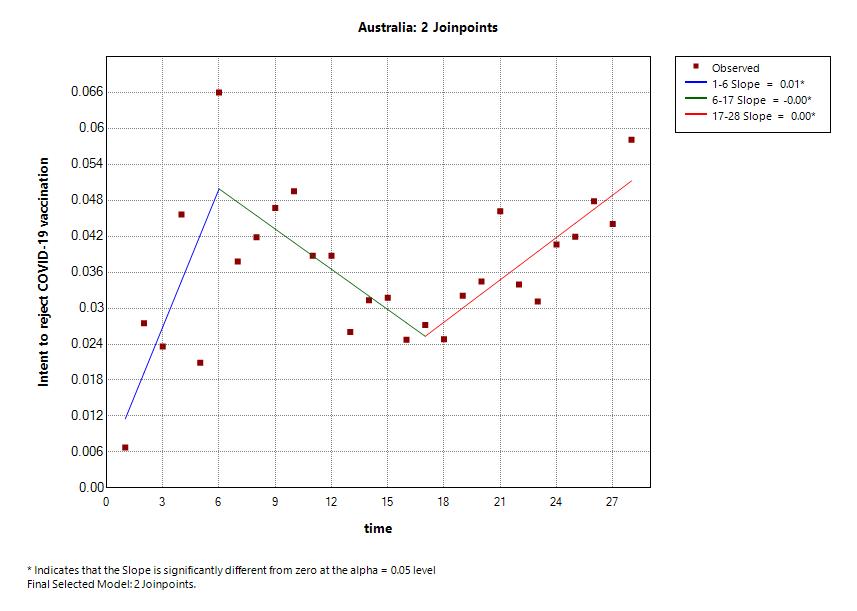


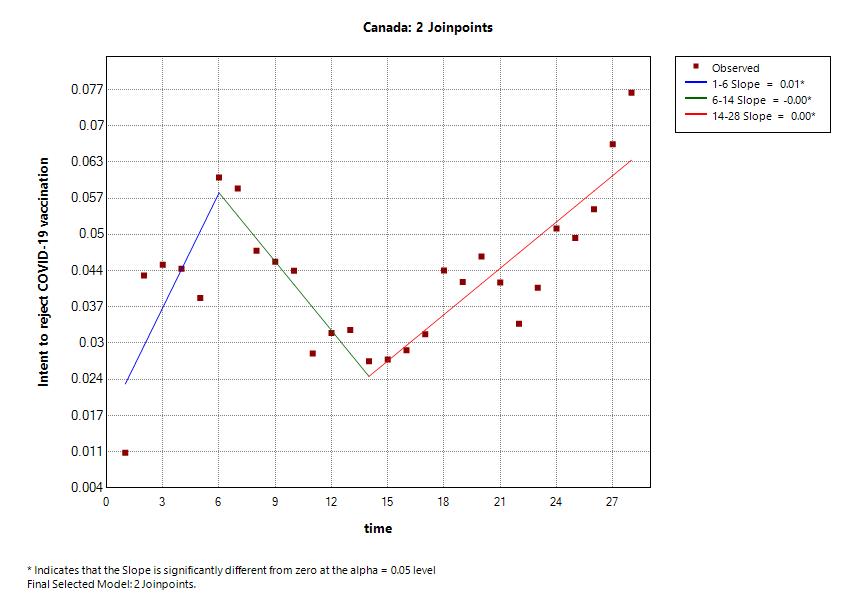

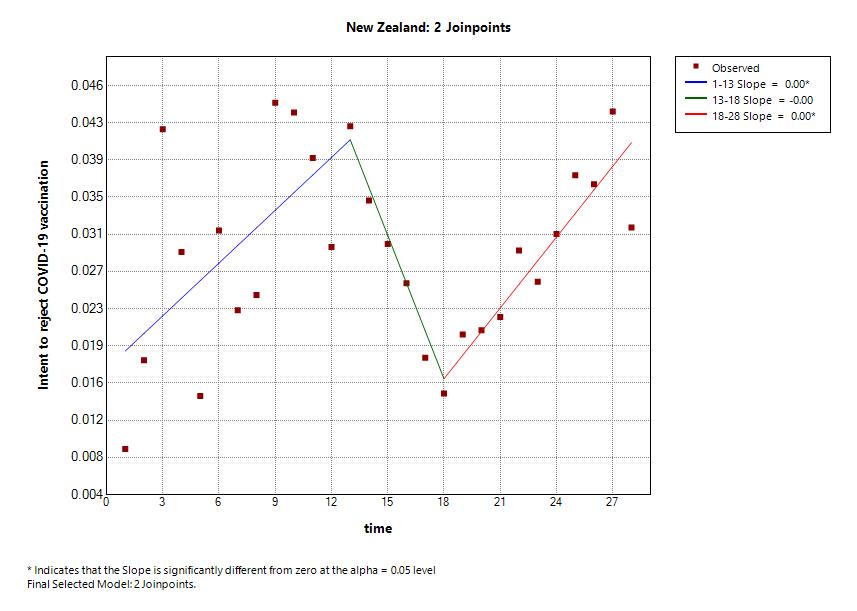


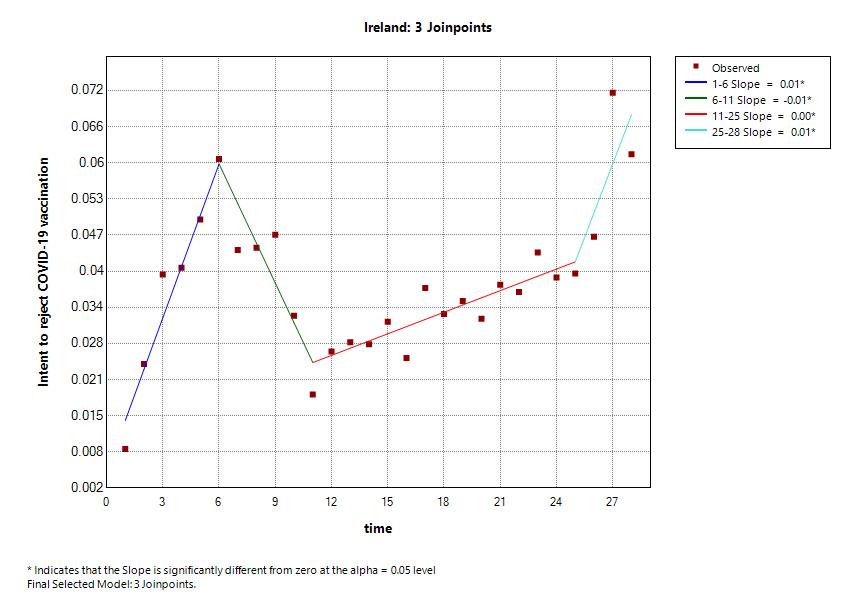


1. Belief that COVID-19 vaccine is effective


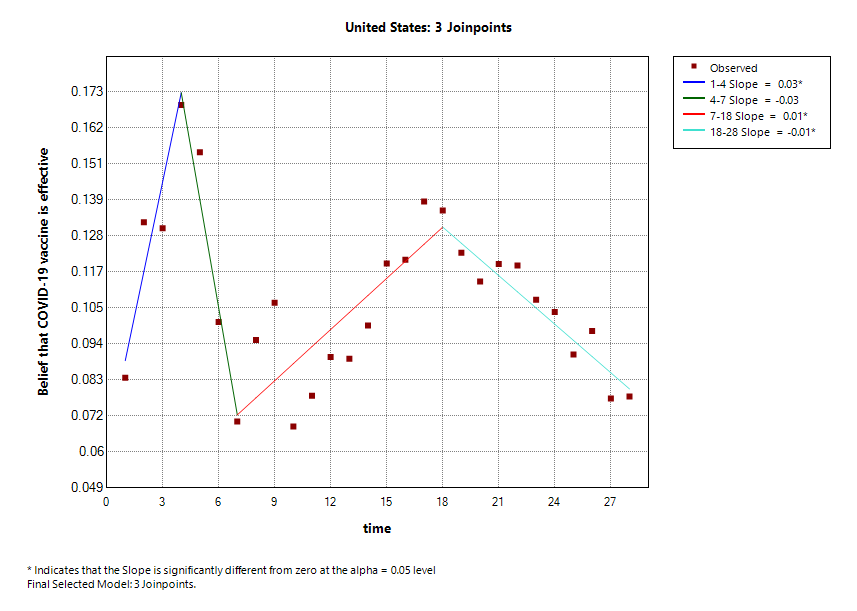


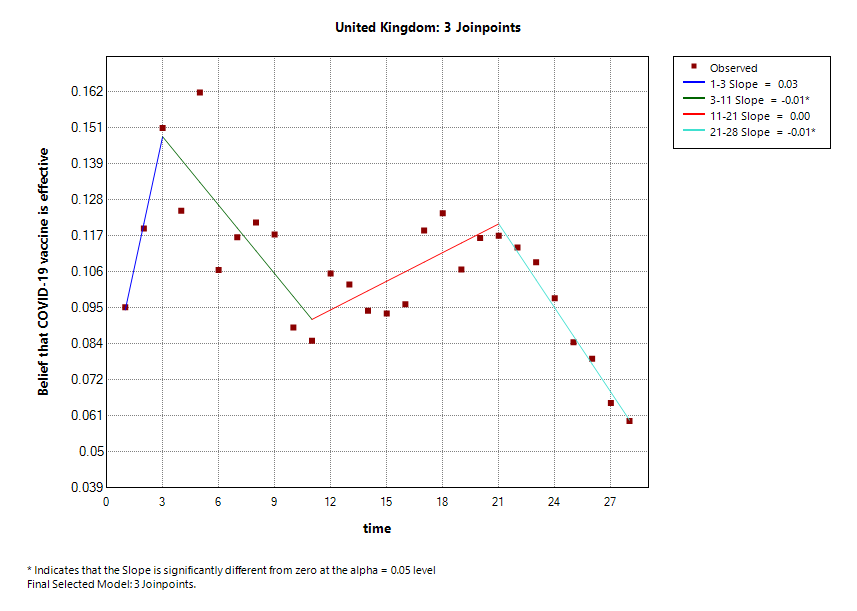


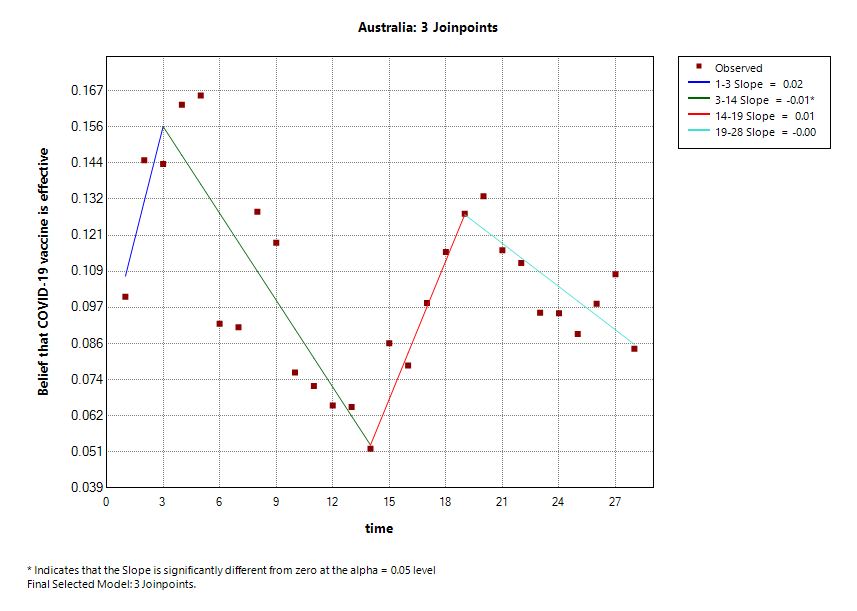


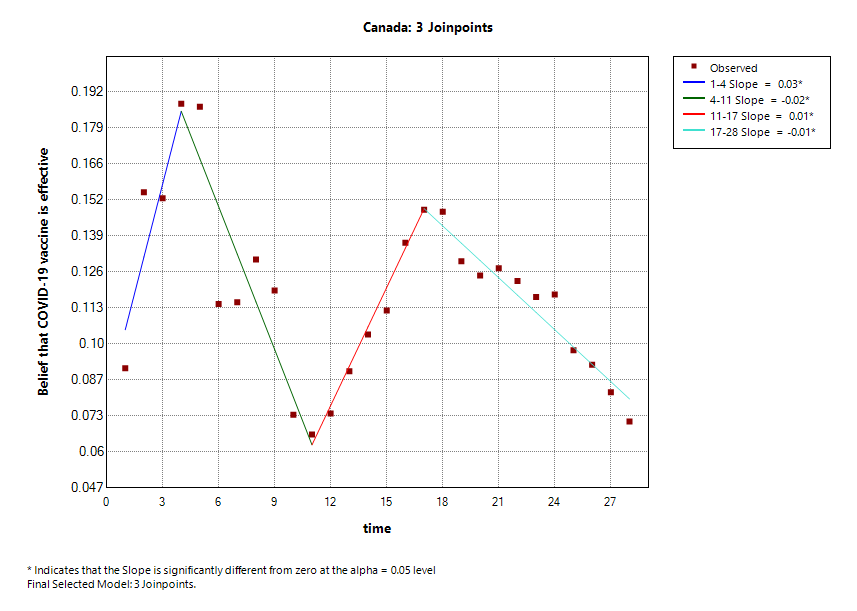


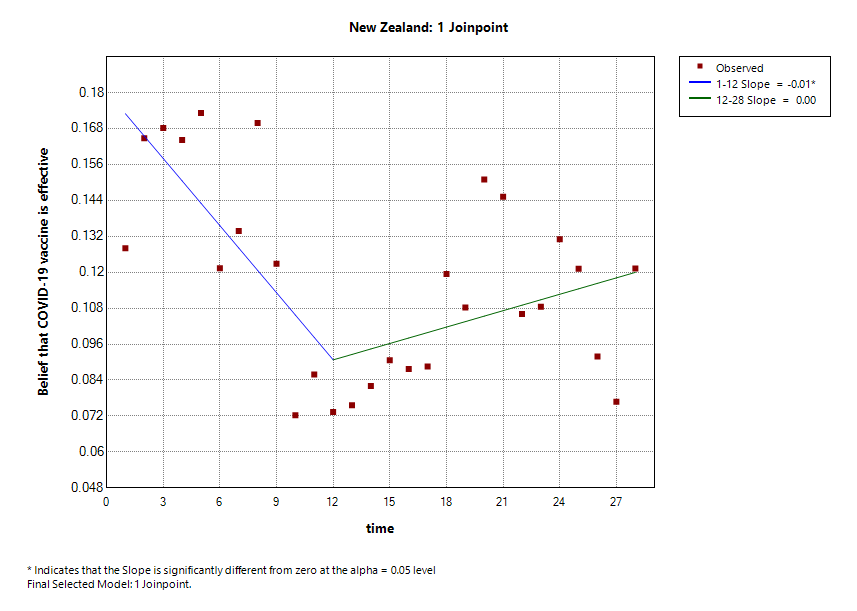


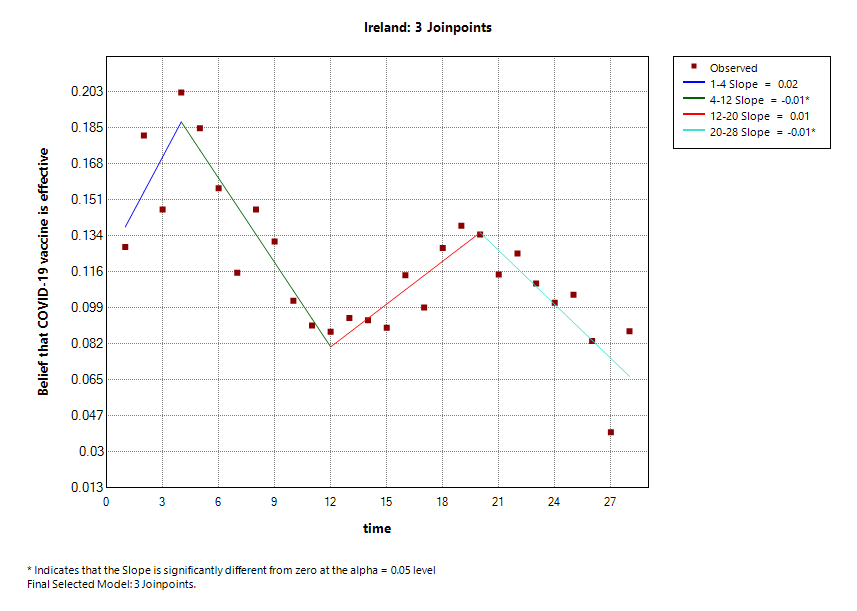


1. Belief that COVID-19 vaccine is not safe


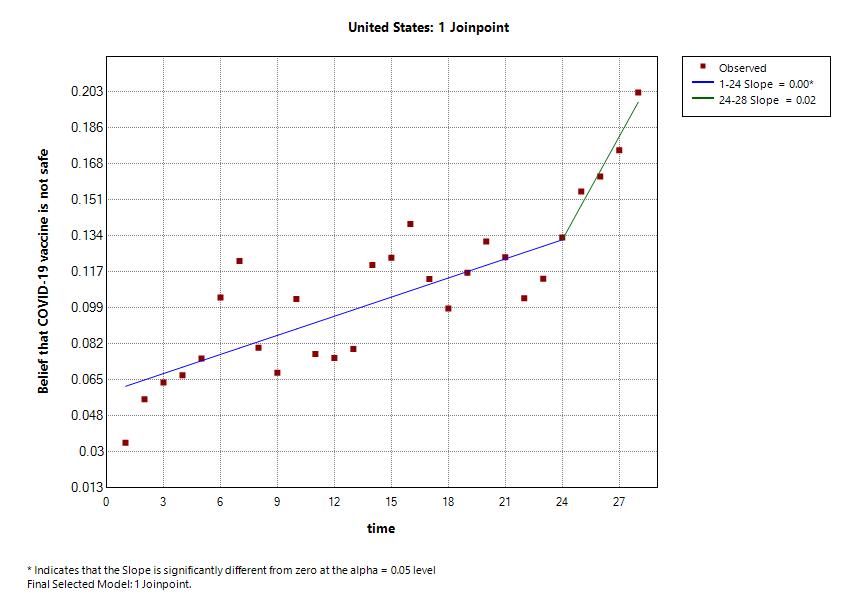


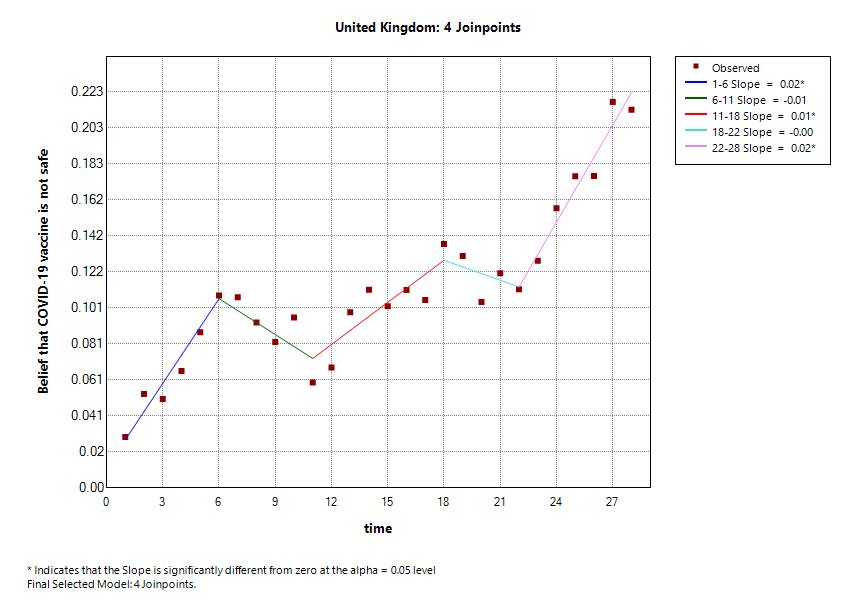


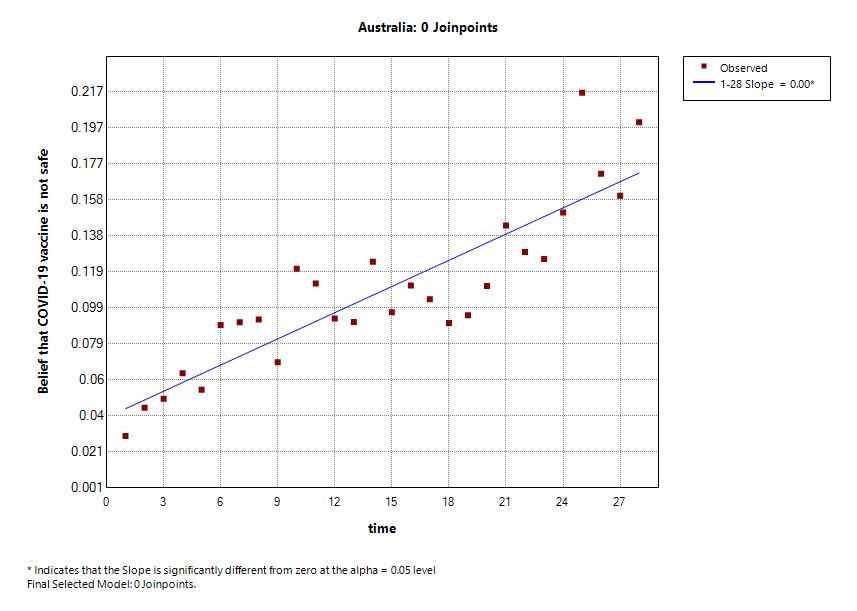


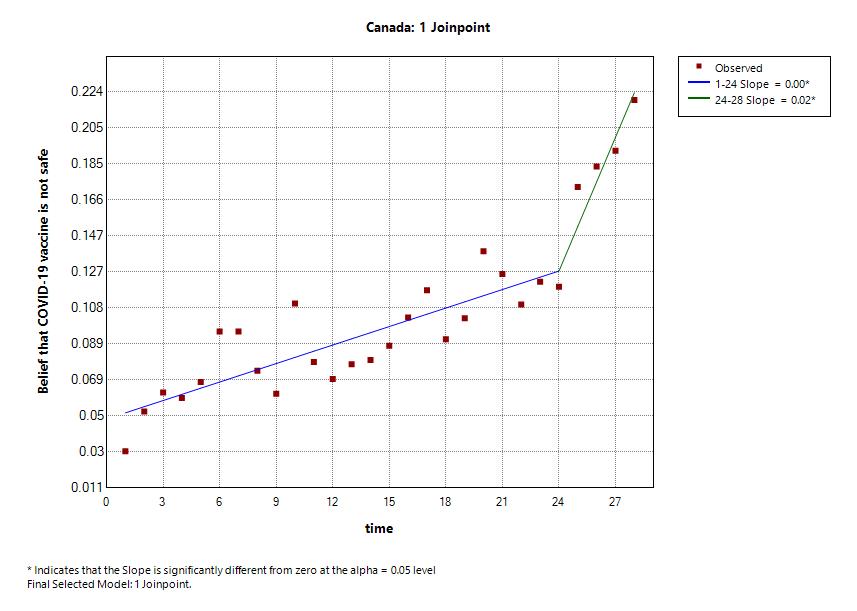


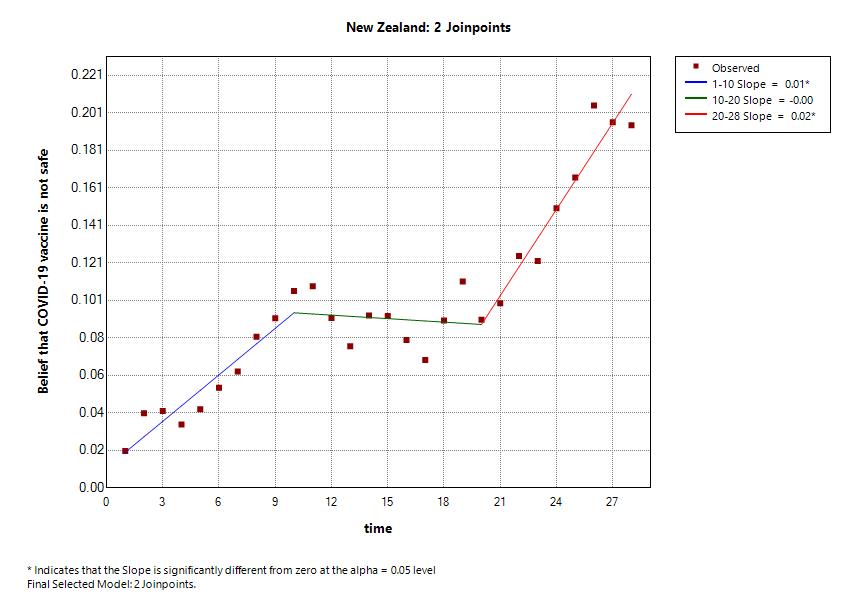


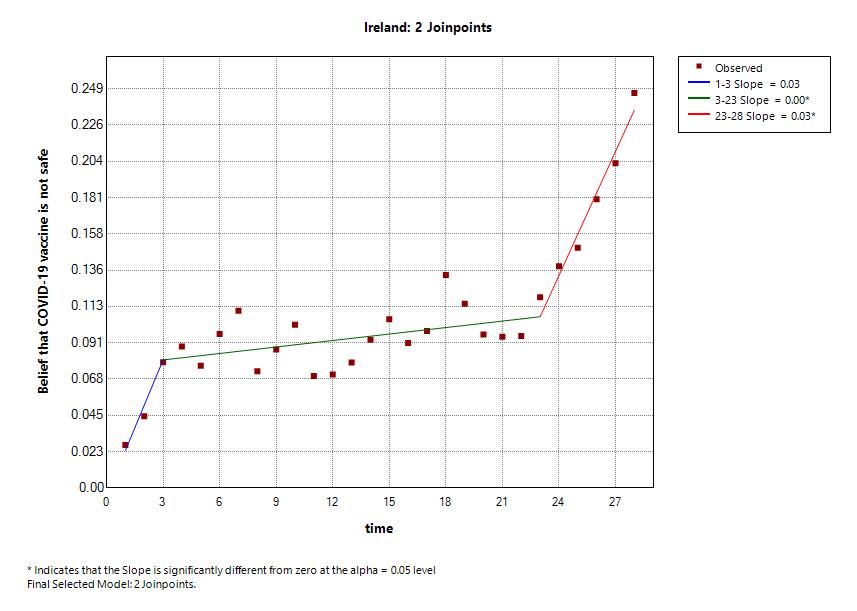

Supplement: Multimedia Appendix 1 [file jmir_v25i1e49753_app1.docx]
